# Comprehensive track-structure based evaluation of DNA damage by light ions from radiotherapy-relevant energies down to stopping

**DOI:** 10.1038/srep45161

**Published:** 2017-03-27

**Authors:** W. Friedland, E. Schmitt, P. Kundrát, M. Dingfelder, G. Baiocco, S. Barbieri, A. Ottolenghi

**Affiliations:** 1 Institute of Radiation Protection, Department of Radiation Sciences, Helmholtz Zentrum München – German Research Center for Environmental Health (GmbH), Neuherberg, Germany; 2 Department of Physics, East Carolina University, Greenville, NC, USA; 3 Department of Physics, University of Pavia, Pavia, Italy

## Abstract

Track structures and resulting DNA damage in human cells have been simulated for hydrogen, helium, carbon, nitrogen, oxygen and neon ions with 0.25–256 MeV/u energy. The needed ion interaction cross sections have been scaled from those of hydrogen; Barkas scaling formula has been refined, extending its applicability down to about 10 keV/u, and validated against established stopping power data. Linear energy transfer (LET) has been scored from energy deposits in a cell nucleus; for very low-energy ions, it has been defined locally within thin slabs. The simulations show that protons and helium ions induce more DNA damage than heavier ions do at the same LET. With increasing LET, less DNA strand breaks are formed per unit dose, but due to their clustering the yields of double-strand breaks (DSB) increase, up to saturation around 300 keV/μm. Also individual DSB tend to cluster; DSB clusters peak around 500 keV/μm, while DSB multiplicities per cluster steadily increase with LET. Remarkably similar to patterns known from cell survival studies, LET-dependencies with pronounced maxima around 100–200 keV/μm occur on nanometre scale for sites that contain one or more DSB, and on micrometre scale for megabasepair-sized DNA fragments.

Radiation therapy is one of the major modalities for the treatment of cancer. While conventional radiotherapy is based on the use of photon beams, irradiation with light ions is getting increasingly popular since dose distributions can be achieved that are better confined to the tumour and spare healthy tissues. In addition, this dosimetric advantage is combined with an increased biological effectiveness of this densely ionizing radiation^1^. Apart from protons, the most frequently used ion species is carbon; however, heavier ions such as oxygen are available in some centres too^2^, and irradiation with lighter ions such as helium or lithium may be advantageous for some tumours^3^.

The optimal use of ion radiotherapy heavily relies on modelling^4^,^5^. The treatment planning has to account primarily for the distribution of the deposited dose. Especially for ions also the increased relative biological effectiveness (RBE) has to be taken into account; clear understanding of RBE effects is essential for optimal use of ion radiotherapy^6^. For protons a generic RBE value of 1.1 is usually taken since the region with significantly enhanced RBE is confined to a sub-mm section at the track ends^7^. For heavier ions, however, the changes of RBE along the beam penetration into tissue cannot be neglected and, therefore, need to be modelled. An important tool used in several clinical centres is the local effect model (LEM)^8^; it predicts the biological effect of ion beams by combining the known response to photon irradiation with an amorphous model of ion track, which describes how the average energy deposition decreases with increasing radial distance from the ion’s path.

A more detailed description of the passage of radiation through matter and its stochastic nature is provided by track-structure simulations^9^. Biophysical simulation tools such as PARTRAC^10^,^11^,^12^ or KURBUC^9^,^13^ follow individual interactions of the primary particle and its secondary electrons with the traversed medium. Further, they account for the subsequent formation of reactive species, their diffusion and mutual reactions, and the induction of damage to cellular DNA and its repair by the cell. Through this, these simulation tools enable assessing biological effects induced by diverse types of radiation on a solid mechanistic basis.

To our knowledge no systematic track structure-based evaluation has been published of DNA damage induced by light ions over the radiotherapy-relevant energy spectrum, i.e. from energies as high as 250 MeV for protons or 400 MeV/u for carbon ions that are needed to achieve the necessary penetration depths in tissue, down to their stopping in the tumour region. However, simulations on the induction of DNA double strand breaks (DSB) by 300 MeV/u carbon beams have been performed^14^ using the Monte Carlo Damage Simulation (MCDS) code, a fast tool that reproduces the results of track-structure studies without explicitly modelling the underlying tracks^15^, which has been recently extended to several types of DNA damage induced by a variety of radiation types including slow heavy ions^16^,^17^. On the other hand, the detailed mechanistic modelling in track structure simulations starts from cross section data that comprise the physics of particle interactions with the traversed medium. For hydrogen and also helium these cross sections have been derived from theoretical considerations and experimental data down to keV energies^18^,^19^,^20^,^21^. For heavier ions, however, track-structure codes are mostly limited to ion energies above 0.3 MeV/u where charge-transfer processes play a negligible role and the ions are mostly bare, since any electrons that might get picked up during the ion’s passage through matter are quickly stripped off again^9^. Recently, a track-structure approach for charge state-dependent carbon ion cross sections including charge-transfer processes has been proposed^22^; however, the model is computationally very expensive, it has not yet been developed for other light ions, and below ~0.5 MeV/u it deviates from the stopping power values recommended by the ICRU^23^,^24^ and SRIM calculations^25^. Commonly, track-structure simulations for ions are based on interaction cross sections obtained by scaling those of protons^13^,^26^; scaling factor is the effective charge squared, determined usually by the Barkas formula^27^. However, at low energies where each slowing-down particle passes through a maximum in the stopping power (the single-particle Bragg peak), this algorithm leads to unrealistically low stopping powers^28^. Therefore, a modification of the scaling procedure has been proposed^28^, which replaces the scaling of proton cross sections by a general effective charge-squared scaling related to hydrogen, including charge transfer processes. It enables track-structure simulations down to energies of ~10 keV/u, where the ion’s residual range in water is only a few tenths of a micrometre, i.e. essentially down to its full stopping. Recently, this achievement has been utilized to investigate the energy-dependent RBE of neutrons^29^,^30^.

In this work, the new scaling approach has been refined by adopting cross section-based fractions of neutral and charged hydrogen states, and it has been validated against standard stopping power data^23^,^24^,^25^; details are provided as Supplementary Information. On this basis, a systematic evaluation with the PARTRAC modules for track structures and DNA damage including the yields of DSB and their complexity is presented for carbon, nitrogen, oxygen, and neon ions, together with a corresponding analysis for hydrogen and helium particles based on established cross-section data^18^,^31^. The study covers the radiotherapy-relevant region of energies, from 256 MeV/u (range in water: 38 cm for protons, 13 cm for carbon ions) down to 0.25 MeV/u (range in water: 4–6 μm) energy when entering the cell nucleus; such very low-energy particles are followed down to their full stopping inside the nucleus. The applicability of the linear energy transfer (LET) concept is discussed that is commonly used in radiation biology and radiotherapy. To deal with the rapidly varying properties of slow ions’ tracks in particular on the distal side of the single-particle Bragg peak, LET values are defined locally and related to the induced DNA damage. The results of this study systematically quantify strand breaks and DSB induced by light ions. As such, these results improve the knowledge on the mechanisms that underpin the biological effectiveness of diverse types of radiation, and may have important implications for radiotherapy with ion beams as well as for radiation protection in space research.

## Methods

PARTRAC^10^,^11^ is a suite of Monte Carlo codes that enables simulations of track structures, the induced DNA damage and its cellular repair following irradiation with photons, electrons, protons and light ions. It integrates experimental and theoretical information on particle interactions with matter, radiation chemistry, DNA and chromatin structure over multiple scales, susceptibility of DNA to damage, and enzymatic reactions that aim at restoring the integrity of the cellular DNA. In this work, PARTRAC has been used to simulate track structures of H, He, C, N, O and Ne ions with energies from 0.25 to 256 MeV/u and the DNA damage induced when hitting nuclei of human lymphocytes. Similar results for human fibroblasts are presented in Supplementary Information.

### Interaction cross sections for ions

In PARTRAC, track structure calculations for light ions and their secondary electrons are performed approximating biological material by liquid water; considering the nuclear environment and explicitly accounting for how the presence of DNA affects the resulting track structures has been found important only for photon irradiation with energies between the carbon and oxygen K absorption edges (0.28–0.54 keV)^32^.

For H and He, the code explicitly traces the individual charge states (H^+^ and H^0^, or He^2+^, He^+^ and He^0^, respectively), using charge state-dependent cross sections^18^,^31^ based on theoretical approaches and extrapolations from experimental data for ionizations, excitations and charge transfer processes (i.e. ionizations upon electron pick-up and emission of a secondary electron upon stripping of the energetic H or He particles). The H and He cross sections implemented in PARTRAC have been derived for energies down to 100 eV; however, its limitation to electronic interaction leads to a practical limit of 1 keV/u, below which nuclear interactions are not negligible.

For ions with atomic number Z > 2, interaction cross sections are determined by scaling the ones for hydrogen, σ_H_, at the same velocity v (i.e., at the same energy per nucleon),


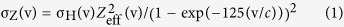


where c denotes the speed of light and Z_eff_ is the effective charge given by the standard Barkas formula^27^:


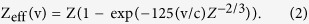


The denominator in Eq. (1) results from the Barkas formula Eq. (2) when it is applied to hydrogen; including this factor in the denominator ensures self-consistence of Eq. (1), i.e. it safeguards that cross sections for all elements including hydrogen scale according to their Z_eff_^2^. At sufficiently high energies, the denominator approaches unity faster than Z_eff_/Z in Eq. (2) for Z > 1, and Eq. (1) equals asymptotically the original Barkas formula Eq. (2); at even higher energies, the scaling simplifies to a Z^2^ law. At low energies, Eq. (1) approaches a minimum value of σ_H_(v) Z^2/3^. Indeed, Eq. (1) corresponds to the stopping power ratios between ions, which exhibit a sigmoidal energy dependence^23^,^33^. Eq. (1) can be viewed as a generalized Barkas scaling law, multiplying σ_H_(v) by a generalized Barkas factor, or, alternatively, as the original Barkas scaling of virtual hydrogen cross sections σ_H_(v)/(1 − exp(−125(v/c))^2^. A scaling scheme that starts from cross sections for helium instead of hydrogen has been adopted in the MCDS^16^,^31^ and compilations of scaling factors^23^,^33^; after refinements analogous to Eq. (1) it may provide better results than the hydrogen-based scaling for particles with electronic structure similar to that of helium.

The Barkas scaling scheme, proposed originally for scaling of the stopping power of ions, is assumed to provide the scaling law for all electronic cross sections, i.e., inverse mean free paths are scaled whereas interaction types, energy transfers and angular distributions are determined as for isotachic hydrogen particles; this is a standard assumption in track-structure studies of irradiation with ions^9^. The novel aspects here are that the inclusion of the denominator in Eq. (1) accounts for self-consistence and, at the same time, extends its validity to much lower energies, and that cross sections for hydrogen (a mixture of protons and neutral hydrogen atoms), σ_H_, are used instead of the ones for protons, σ_p_, adopted in earlier studies.

The actual charge states of the traversing ion are not followed explicitly. In the course of its slowing down, the ion is characterized by its gradually decreasing effective charge. Details on the fractions of charge states of hydrogen and the determination of hydrogen cross sections used in this work for scaling are reported in Supplementary Information. Moreover, a validation of this scaling scheme regarding its self-consistence and the resulting stopping powers is provided in Supplementary Information.

### Initial DNA damage

Track structures, i.e. spatial distributions of energy deposition events, have been calculated for the considered ions and energies in liquid water as an approximation for the traversed cellular medium. For hydrogen and helium ions, charge state-specific cross sections^18^,^31^ have been used; cross sections for heavier ions have been based on the above-described scaling from those of hydrogen. All secondary electrons have been followed down to 10 eV energy using electron cross sections by Dingfelder *et al*.^34^. Nuclear interactions have not been considered; only electronic interactions of primary ions and secondary electrons have been followed.

Standard PARTRAC methodology assessing DNA damage has been used^10^. Briefly, DNA damage due to direct effects of radiation has been determined by superposing a multi-scale DNA target model with the distributions of energy depositions from the physical stage. Strand breaks have been assumed to occur with a probability that increases linearly from zero at 5 eV to unity at 37.5 eV energy deposit in a volume of a single sugar-phosphate group; this approach^32^ accounts for experiments on strand break induction by low-energy (5 eV) electrons^35^. Since these pioneering findings, the research on low-energy electrons has considerably evolved^36^. Experiments with plasmid DNA under ultra-high vacuum conditions have shown that low-energy electrons damage DNA directly by forming transient negative ions of DNA subunits that decay via dissociative electron attachment channels, and indirectly by creating reactive species from nearby water molecules. However, such experimental conditions differ from the molecular environment of the cell^37^. Due to the lack of data on low-energy electron processes under cell-like conditions, these processes have not been explicitly modelled in this work. Nevertheless, the adjustment of strand break induction parameters to experimental DNA damage yields implicitly includes to some extent effects by low energy electrons, since they do contribute to the total DNA damage for any radiation quality.

To account for indirect effects of radiation within the subsequent pre-chemical stage, all energy deposition events outside the DNA target volume and the histones, but still in their vicinity, have been converted to reactive species. For very densely ionizing particles, i.e. at high LET values, especially in the central dense part of the track (track core) the spatial density of individual energy deposition events may get so large that more than a single ionization and/or excitation would occur in a single water molecule. As interactions leading to doubly ionized molecules are not included in the PARTRAC model of the physical stage, additional energy deposits to an already ionized or excited water molecule have been shifted to its neighbours (“delocalization”) and subsequently processed by the standard pre-chemical and chemical modules that also work only with single ionizations^38^.

The formed radical species have been traced step-by-step during the subsequent chemical stage from 10^−12^ s up to 10^−7^ s in their diffusive motion and reactions^38^, taking into account the impact of the structure of the DNA and the histones^39^. Interactions of hydroxyl radicals (^•^OH) with DNA constituents were scored as indirect effects of the studied radiation. The DNA volume has been determined by the union of the atomic spheres, with van-der-Waals radii doubled in order to account for a surrounding hydration shell and to remove holes inside the DNA structure; van-der-Waals radii have been multiplied by 3 for histones, since their atomic representation^32^ does not include hydrogen atoms. Strand breaks from indirect effects have been assumed to arise from 65% of interactions between ^•^OH radicals and the deoxyribose moiety of DNA.

A spherical human lymphocyte nucleus with 10 μm diameter has been modelled in PARTRAC by a randomized walk on a 50 nm grid, using five types of stackable chromatin fiber elements of 50 × 50 × 50 nm^3^ with atomic representation of the 5–6 kbp of DNA and histones inside^40^. Chromatin fiber loops of ~100 kbp have been generated between nuclear attachment sites; then, multiple loops have formed a 1 Mbp chromatin domain, and finally between 50 and 245 such domains have built up the chromosomes^10^,^40^.

### DSB induction and DNA damage classification

Although base lesions are scored in PARTRAC too^10^, this work is focused on the induction of DNA strand breaks (SB), in particular DNA double-strand breaks (DSB) and their clustering, since it is DSB that are commonly considered as lesions most relevant for biological effects of ionizing radiation. Inclusion of results on base damage would go beyond the scope of this work; its biological importance, especially in the framework of DSB and non-DSB clustered lesions, is well known and accepted^41^,^42^. DSB are formed by pairs of SB on opposite strands with not more than 10 bp distance. To account for radical transfer and further experimental results, an additional 1% conversion probability from an SB to a DSB has been assumed for both directly and indirectly induced breaks^32^.

To deal with strand break patterns that are formed when chromatin is crossed by a slow ion with very high LET values (up to 1,500 keV/μm for Ne ions), a refined scoring scheme for the DNA damage induced by single tracks has been adopted in this work. It should be noted that details of the scoring scheme have some impact on the resulting DNA damage yields^43^. All *strand breaks (SB*) have been divided into *double-strand breaks (DSB*), i.e. pairs of SB on opposite strands with not more than 10 bp distance and 1% converted SB, and the remaining category of *single-strand breaks (SSB*) including SB in vicinity of DSB. Multiple SB are scored individually only if there is at least one unbroken sugar-phosphate group between them, i.e. breaks on directly adjacent nucleotide pairs are merged into a single one. DSB pairs not separated by more than 25 bp have been merged to *DSB clusters*; these are equivalent to dsb++ in the notation by Nikjoo *et al*.^13^,^44^. DSB clusters may include more than two DSB and thus more than a single DNA fragment of not more than 25 bp length. The mean number of DSB in DSB clusters is referred to as *DSB multiplicity*; it is close to 2 for LET values below ~200 keV/μm (cf. Results). Finally, *DSB sites* are introduced as a quantity for DSB induction on a non-local (>10 nm) scale. The number of DSB sites includes the number of isolated DSB, i.e. DSB not being in a DSB cluster (dsb0 and dsb+ in the notation by Nikjoo *et al*.^13^,^44^), and the number of DSB clusters. This scheme serves as a means of characterizing the overall chromatin breakage as well as local DSB complexity. It addresses important differences in the damage pattern: for example, 7 DSB can be distributed as isolated ones over the nucleus (7 DSB sites, 0 DSB clusters) after low-LET irradiation, or as 2 DSB clusters (both with DSB multiplicity 2) and 3 isolated DSB, in total 5 DSB sites, after high-LET irradiation, or as a single DSB cluster with DSB multiplicity 7 (also scored as a single DSB site) at highest LET values near the Bragg peak.

To quantify the distribution of DNA damage on larger scales, single-track DNA fragments have been scored in various size intervals; results for 60–100 bp, 0.3–3 kbp, 10–100 kbp and 0.3–3 Mbp are presented. These fragments correspond to pairs of DSB sites induced by a single track when it crosses the major elements of chromatin structure in human cell nuclei: a nucleosome, a chromatin fiber, a chromatin fiber loop, and a chromatin domain (or a giant loop), respectively. The results are reported in terms of their yields per unit dose; however, DNA fragments in general do not scale linearly with dose since an additional break due to another track may create two shorter fragments from a longer one. Thus, in particular the results for the two larger fragment size intervals refer to a low-dose situation (below about 0.1 Gy) where the contribution of fragments from DSB due to different tracks is below 10% for all investigated radiation qualities.

### Simulated irradiation setup

**Figure 1:**
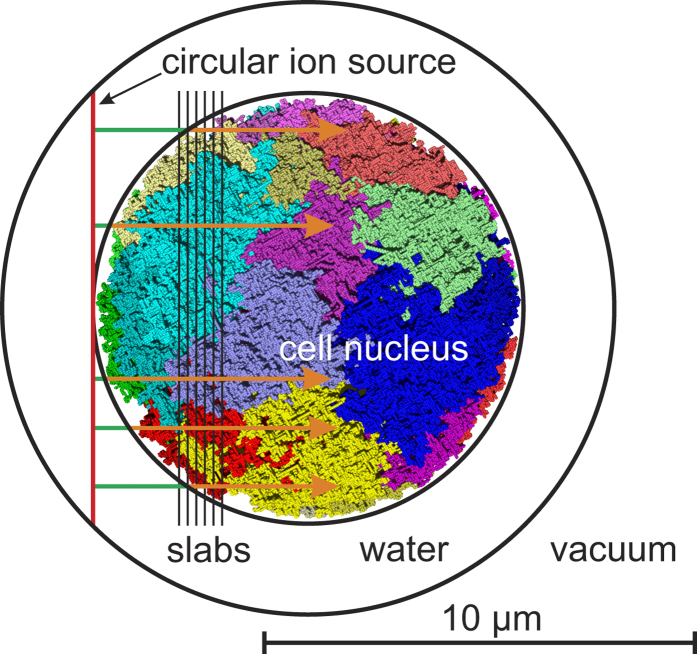
Setup of irradiation simulations. A circular ion source (red) is located tangentially to the cell nucleus in a surrounding water shell. 5 particles per run start with a given initial energy from random positions on the source area in normal direction to it; arrows illustrate carbon ion tracks of 0.25 MeV/u initial energy (range ~6 μm). Determination of LET (Fig. 2a) is based on energy deposits including secondary electrons inside nuclear volume (orange arrows); energy deposited outside the nucleus (green parts of the arrows) is not considered. Local analysis of DNA damage by slow ions (Fig. 6) is based on DNA amount and particle stopping power (Fig. 2b) in 200-nm-slabs (thin black lines). Primary and secondary particles leaving the surrounding shell are no longer scored. Coloured structures of the cell nucleus refer to chromatin of different chromosomes.

The setup of the simulation calculations is presented in Fig. 1. Simulations have been performed for nuclei of human lymphocytes irradiated by ^1^H, ^4^He, ^12^C, ^14^N, ^16^O, and ^20^Ne particles at initial (starting) energies of 0.25, 0.5, 1, 2, 4, 8, 16, 32, 64, 128, and 256 MeV/u. The source of these ions was an 80 μm^2^ circle tangential to the cell nucleus. Per simulation run, ions have been started exactly parallel from 5 random positions on this circle, in perpendicular direction to it; this corresponds to the fluence of 0.0625 μm^−2^ per run. To reach sufficient statistics, 1024 to 4096 runs for H and He ions and 256 runs for C, N, O, and Ne ions have been performed for each energy value. For each run, DNA damage yields have been determined; the presented results are the mean values of all runs and the errors are twice the standard error of the mean. The cell nucleus was modelled as a sphere with 10 μm diameter. At the beginning of each run the source has been rotated by a randomly drawn angle in space to avoid artefacts that may otherwise arise from the alignment of tracks with chromatin model structures. The interactions of primary particles and secondary electrons were scored inside a sphere filled with liquid water with 14.22 μm diameter concentric to the nuclear target volume which safeguards the inclusion of the rotated source area.

This setup with a source directly touching the spherical nucleus has been chosen in order to limit the thickness of material that low-energy ions with a short range have to pass through; e.g. 0.25 MeV protons penetrate only about 4 μm in water, and hence even with this setup they stop before reaching the cellular mid-plane. Using the same geometrical setup also for ions at high energies has reduced the computational expensiveness of the simulations. However, it is important to note that the adopted setup does not provide an electronic equilibrium, in particular for higher ion energies. The results are compatible with experiments in which a layer of cells is irradiated with a negligible material penetration before the ions enter the nuclear volume.

### Characterizing ion tracks by LET

Linear energy transfer (LET) is a local quantity widely used to characterize the radiation quality^45^,^46^. Mean LET values are appropriate for track segments over which the characteristics of the studied radiation (especially particle energy) do not change significantly. This is the case for high-energy ions which lose only a marginal part of their energy when passing through a cell nucleus. However, energy loss becomes critical for low-energy ions, in particular for very low-energy ones (‘stoppers’) that actually stop inside the cell nucleus.

To deal with this issue, in this work the LET has been calculated by the classical relation with dose D and fluence φ as a mean value inside the cell nucleus for ions with energy sufficient to pass through it (actually 1 MeV/u and more),


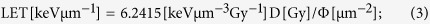


the numerical factor arises from the conversion of units in a medium with the density of liquid water (1 g cm^−3^). The dose has been obtained from all energy deposition events by the primary and all its secondary particles that occurred within the volume of the model nucleus. By this definition, the LET used here is analogous to the concept of the dose-mean lineal energy in microdosimetry^47^. The LET definition used here also bears analogies to the concept of restricted LET (L_∆_), in which only secondary electrons with energy up to ∆ are included, or even more the spatially restricted LET (L_r_) that limits the position of energy depositions to a cylinder with radius r around the track^21^. L_∆_ has been proposed as a means that correlates with biological effects better than the unrestricted LET does^48^. For the sake of brevity, only the term “LET” is used throughout this work for the above-mentioned LET definition; note that it does not correspond to electronic equilibrium conditions due to the position and size of the source (see below). For high-energy ions the LET differs considerably from the ion’s stopping power, which is defined by the energy loss of the particle.

For ions with lower energies, which cannot pass through the whole nucleus, the volume to which the energy deposition has been related in the dose determination has been reduced to the spherical cap whose height is given by the ion’s range in water. The LET has then been calculated from Eq. (3) again as a mean value for the irradiated part of the nucleus. In this case, the difference between the energy imparted to the medium and that lost by the particle is minimal, since the secondary electrons possess short ranges only. The LET is thus practically identical to the track-averaged stopping power, averaged over the track part within the nucleus (orange parts of the arrows in Fig. 1) and over tracks that hit the nucleus, centrally as well as peripherally. To avoid the uncertainties related to ion slowing down at lowest energies, the range has been determined by extrapolating to zero energy the depth at which the ion’s energy decreased to 10% of its initial value.

For a detailed study of DNA damage for ion energies around and below their maximum stopping power, the results obtained for 0.25 MeV/u initial energy have been differentially analysed in dependence on the distance from the source. To this end, LET values have been determined locally from the energy deposited in a stack of 50 slabs of 200 nm thickness (a few slabs illustrated by thin black lines in Fig. 1) and corresponding dose values were calculated for each slab according to Eq. (3). The yields of DNA damage per slab are reported relative to the actual DNA amount, which varies among the slabs. To avoid potential issues due to the alignment of the slab geometry and the chromatin model, the depth-dependent amount of DNA per slab has been determined by averaging over numerous rotations of the model nucleus.

### Characterizing ion tracks by (Z_eff_/β)^2^

DNA damage yields are presented also as a function of the square of the effective charge divided by the velocity (Z_eff_/β)^2^ which is another commonly used parameter for radiation quality^49^ that may provide a lower dependence on the particle type than the LET. For the sake of consistency with the MCDS and other published data, the Barkas formula in Eq. (2) has been used for the determination of Z_eff_; the general results do not change upon using its division by the corresponding term for hydrogen according to Eq. (1). Values of (Z_eff_/β)^2^ have been determined for the mean energy inside the nucleus, taking into account the mean energy loss during transport of the particles into the nucleus. For comparison of the LET and (Z_eff_/β)^2^ concepts, the values of (Z_eff_/β)^2^ and of the LET resulting from dose and fluence according to Eq. (3) for these mean energy values are presented in Supplementary Fig. S6.

### Comparison with MCDS results

In the absence of experimental or track-structure based modelling studies on DNA damage induced by low-energy ions, the present PARTRAC results have been compared with published data from the Monte Carlo damage simulation (MCDS) tool^15^,^16^,^17^. The MCDS uses an ad-hoc procedure to determine the spatial clustering of lesions within DNA: Elementary DNA lesions (1300 strand breaks and 3900 base lesions per Gy per cell) are distributed at random over a relatively short DNA segment, and the resulting damage pattern is classified into clusters separated by at least 9 bp of intact DNA. The reported parameters are assumed to be independent of radiation quality, but the DNA segment length (<150 kbp) to depend on (Z_eff_/β)^2^, which is considered as the parameter describing radiation quality. The MCDS input parameters were fitted so as to approximate the results of track-structure simulations for electrons, protons and alpha particles^15^. The MCDS algorithm is much quicker than these track-structure simulations, since it avoids event-by-event modelling of tracks and subsequent processes that lead to DNA damage. The most recent MCDS version covers ions up to iron with 1 ≤ (Z_eff_/β)^2^ ≤200,000 and a variety of DNA damage classes induced under normoxic or hypoxic conditions^17^. The simulated damage within the small DNA segment is scaled to the total amount of DNA in a cell; this is applicable to a uniform irradiation setup. For non-uniform irradiation, the MCDS simulations performed in small sub-regions of cell nucleus with approximately uniform irradiation would have to be combined^14^,^17^.

## Results

### Characterizing ion tracks by LET

**Figure 2:**
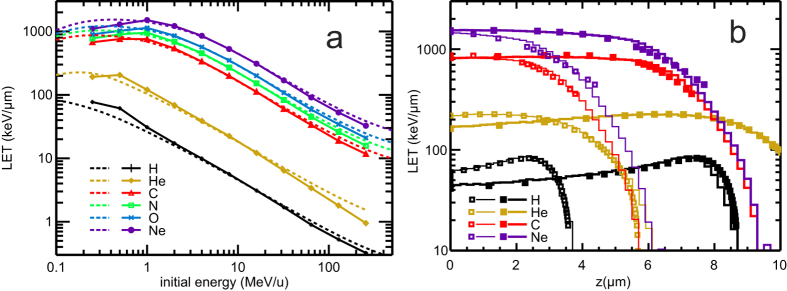
LET in dependence on the initial particle energy and penetrated depth. Panel a: LET calculated from energy deposited within the cell nucleus (symbols connected by solid lines to guide the eyes) and comparison to stopping powers from ICRU^24^,^50^ (dotted lines). Panel b: LET determined from energy deposited in 200 nm slabs perpendicular to particle transport direction (histograms) for ions with 0.5 MeV/u (thick lines) or 0.25 MeV/u initial energy (thin lines). Stopping power data from ICRU^24^,^50^ shown by symbols (0.5 MeV/u: filled squares, 0.25 MeV/u: empty squares).

In Fig. 2a, the LET values calculated from energy deposits within the spherical model nucleus are plotted for the studied ions and energies (symbols connected by solid lines); here and in the following figures, the results for H are depicted by black, He by yellow, C by red, N by green, O by blue, and Ne by violet symbols and/or lines. For the two lowest energies, ions’ ranges are lower than the diameter of the nucleus, and hence only the spherical cap with this height has been considered in calculating LET values. At the highest energies, the ion’s LET, being defined by the energy imparted to the target volume, is lower than the particle’s stopping power, defined by the energy lost by the particle (dotted lines, ICRU data^23^,^24^,^50^). This difference is due to the setup used here, with the source directly attached to the target volume, so that electron equilibrium has not built up yet. A significant part of the energy lost by the ion is carried by secondary electrons with relatively high energies and is deposited laterally and/or at greater penetration depths outside the nucleus. Such energy deposits are not scored within the LET definition used here; consequently, the LET is lower than the stopping power, by about 20% for 256 MeV/u ions (Fig. 2a). This effect gets smaller as the ion’s initial energy decreases, since the liberated secondary electrons are limited to smaller energies and hence shorter transport distances then. For the lowest energies, the build-up occurs over very short distances and virtually no energy is transferred outside the nucleus, and consequently the LET closely matches the stopping power. However, the stopping power data in Fig. 2a refer to initial ion energies, whereas the LET values are averaged over track segments within the nucleus. In the low-energy region, the stopping power significantly varies in the course of the ion’s passage through the cell nucleus. For instance for carbon ions emitted from the source with 0.25 MeV/u, the stopping power equals its maximal value, 870 keV/μm according to ICRU data^24^, and then decreases with increasing penetration depth. The LET calculated from energy deposits in the nucleus and averaged over the ion’s range (3.8 μm according to ICRU data^24^) is about 790 keV/μm. Such differences between the ICRU stopping power and the average LET get smaller if the LET is related to the mean value of the particle energy inside the nuclear volume (Supplementary Information).

To capture the rapidly varying stopping power along the penetration depth of low-energy ions, LET values have been locally determined by scoring Monte Carlo-simulated energy deposits within 200 nm slabs of the cell nucleus. For H, He, C and Ne ions with 0.25 or 0.5 MeV/u energy, these LET values are plotted in dependence on the penetration depth in Fig. 2b; results for N and O are quite similar to C and Ne and have been omitted for the sake of better visibility. The usual averaged LET values basically correspond to approximating these local LET distributions by single values over the whole ions’ ranges. The LET values in Fig. 2b characterize the region around and the distal branch of the single-particle Bragg peak. The results are found in excellent agreement with corresponding ICRU stopping power data^24^,^50^ (square symbols). Nevertheless, it should be noted that these low-energy regions are subject to large uncertainties, in particular below the low energy limit 25 keV/u of the ICRU data^24^ for ions heavier than He.

### LET-dependent DNA strand breakage

**Figure 3:**
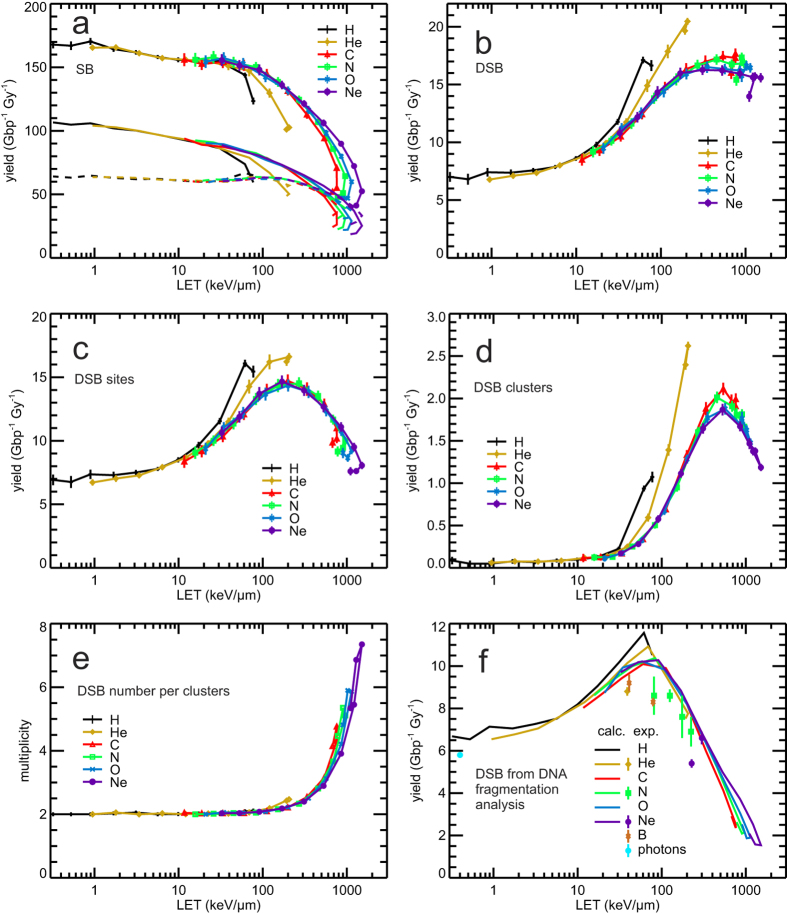
Ion irradiation-induced DNA damage in dependence on LET. In panel a–e, simulation results are shown by symbols connected by lines to guide the eyes; error bars in panel a–d denote two standard errors of the mean (~95% confidence intervals) of the performed simulations. Panel a: SB yields from direct effects (dashed lines), indirect effects (dash-dotted lines), and both effects (symbols and solid lines). Panel b: DSB yields, in which isolated ones as well as individual DSB in DSB clusters are scored. Panel c: Yields of DSB sites, which comprise isolated DSB and DSB clusters; a DSB cluster is scored as one DSB site. Panel d: Yields of DSB clusters; a cluster includes two or more DSB within not more than 25 bp distance. Panel e: Average multiplicity of DSB in a cluster. Panel f: DSB induction by ^60^Co γ-rays, He, B, N and Ne ions measured by the pulsed-field gel electrophoresis technique from DNA fragments sized between 5 kbp and 6 Mbp^52^,^53^ (symbols) and corresponding simulation results considering detectable DNA fragments (solid lines).

The PARTRAC biophysical tools have been used to simulate ion-induced DNA damage in terms of strand breaks (SB), double-strand breaks (DSB) and its clustering on nanometre and micrometre scales in spherical human lymphocyte nuclei. In Fig. 3, DNA damage yields per unit dose and unit amount of DNA (Gy^−1^ Gbp^−1^) are shown in dependence on LET. In Fig. 3a, the calculated SB yields (symbols connected by lines) are presented together with the contributions from direct effects (through direct energy depositions within the DNA and its hydration shell; dashed lines) and from indirect effects (due to attacks of ^•^OH radicals from water radiolysis upon energy deposition outside the DNA; dash-dotted lines). The total (i.e., direct plus indirect) SB yields for different ions are quite similar and follow a unique dependence on LET up to about a third of the ion’s maximal LET value with a slightly decreasing trend from 170 to 150 Gy^−1^ Gbp^−1^; from there on, an ion-specific drop in SB yields occurs with further increasing LET. At low and medium LET, the majority of SB arises from indirect effects (more than 60% of SB at 1 keV/μm); at high LET, direct effects become dominant. The yields of indirect SB decrease with increasing LET, from ~100 Gy^−1^ Gbp^−1^ at the lowest LET values down to values as low as 20 Gy^−1^ Gbp^−1^ for 0.25 MeV/u neon ions. The directly induced SB remain constant, ~60 Gy^−1^ Gbp^−1^, up to LET values ~300 keV/μm, and then also decrease with increasing LET, down to ~30 SB per Gy and Gbp for the ions’ highest possible LET values. The decrease in indirect effects with increasing LET arises due to mutual reactions between the products of water radiolysis in dense tracks of low-energy ions, leaving less radicals to attack the DNA. The decrease in SB from direct effects at very high LET follows from depositing energy to the DNA molecule (or its hydration shell) at a density that is higher than what is needed for its breakage; the extra energy is thus wasted in a kind of overkill effect. At energies below the maximal LET values, on the distal branch of the single-particle Bragg peak, the ions are less effective than at the same LET on the proximal branch of the single-particle Bragg peak; thus, when plotted in dependence on the LET, hooks appear around its maximal values.

The simulation results for the induction of DSB are presented in Fig. 3b; shown are LET-dependent yields of DSB, including both clustered DSB (for which each DSB is scored individually) and isolated ones. As by the SB, also the DSB yields largely follow a unique dependence on LET, indicating that LET as defined in this work is a useful quantity characterizing the radiation quality. DSB yields generally increase with increasing LET, from ~7 Gy^−1^ Gbp^−1^ at 1 keV/μm, up to saturation by 16–17 Gy^−1^ Gbp^−1^ at 300–500 keV/μm. At high LET values, however, ion-specific behaviour occurs. In particular, at a given LET, hydrogen ions are the most effective species in inducing DSB, followed by helium ions; e.g. at 60 keV/μm, H ions induce ~17, He ~14, and heavier ions ~12 DSB per Gy and Gbp.

When multiple DSB in a DSB cluster are scored as a single DSB site (an isolated DSB also being a single DSB site), the simulation results (Fig. 3c) show patterns known from radiobiological studies^51^, in which ion specific peaks in the RBE for cell killing are usually observed in the range from 100 to 200 keV/μm. The calculated yields of DSB sites upon irradiation with ions gradually increase with increasing LET, reach a maximum at ~200 keV/μm where the yields are about twice higher than at the lowest LET values, and then drop to values comparable to those at low LET. Again, hooks are present around the highest LET values. Importantly, above ~10 keV/μm helium and especially hydrogen ions are more effective than heavier ions at the same LET.

The yields of DSB clusters (Fig. 3d; defined as groups of 2 or more DSB not separated by more than 25 bp from each other) and their multiplicity (i.e. the mean number of DSB per DSB cluster; Fig. 3e) increase in a rather pronounced way with increasing LET. DSB clusters are produced most effectively (1.8–2.1 Gy^−1^ Gbp^−1^) by particles heavier than He at ~500 keV/μm; for He, the maximum is ~2.5 Gy^−1^ Gbp^−1^ at ~200 keV/μm and for hydrogen ~1.1 Gy^−1^ Gbp^−1^ at ~80 keV/μm. These efficiencies in DSB cluster induction are twenty- to fiftyfold higher than those at low LET (see Supplementary Information on RBE). At the highest LET values (lowest energies), the DSB cluster yields decrease, but remain significantly higher than those at low LET. At low LET, only a few DSB clusters are formed, consisting nearly always of a single DSB pair. With increasing LET, the mean DSB multiplicity per cluster and, hence, the nanometre-scale complexity of the DNA lesion increase, up to on average almost 5 DSB per cluster for 750 keV/μm C or more than 7 DSB per cluster for 1500 keV/μm Ne ions (Fig. 3e).

In Fig. 3f, the present simulation results have been compared with experimental data on DSB induction in fibroblasts exposed to ^60^Co γ-rays, He, B, N and Ne ions^52^,^53^. These data were obtained by DNA fragment analysis with the pulsed-field gel electrophoresis technique by measuring fragments with sizes between 5 kbp and 6 Mbp. Since DNA damage yields and initial fragmentation in fibroblasts and lymphocytes are very similar^40^ (see Supplementary Information), we show simulation results for lymphocytes in the comparison. In absolute terms, the simulations on pairs of DSB sites that produce DNA fragments of this size interval (lines) overestimate the measured yields (symbols with error bars) by about 10–20% only. In terms of RBE, the simulations do reproduce the measured trends in LET-dependent DSB yields almost perfectly (Supplementary Information).

### DNA strand breakage in dependence on (Z_eff_/β)^2^

**Figure 4:**
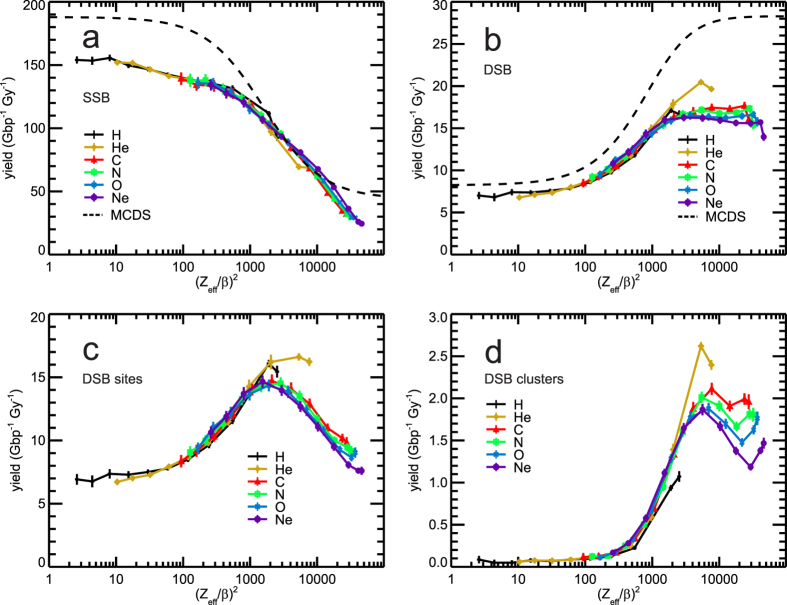
Ion irradiation-induced DNA damage in dependence on (Z_eff_/β)^2^. Simulation results are shown by symbols connected by lines to guide the eyes; error bars denote two standard errors of the mean (~95% confidence intervals) of the performed simulations. Panel a: SSB, i.e. breaks in DSB are not included (symbols and solid lines), with prediction of MCDS^17^ (dashed line). Panel b: DSB yields (symbols and solid lines) with prediction of MCDS^17^ (dashed line). Panel c: Yields of DSB sites, which comprise isolated DSB and DSB clusters; a DSB cluster is scored as one DSB site. Panel d: Yields of DSB clusters; a cluster includes two or more DSB within not more than 25 bp distance.

In Fig. 4a, the yields of single-strand breaks (SSB) are presented for the six particle types (solid lines in colours) together with a prediction according to the MCDS^17^ (black dashed line). Compared to the results for SB in dependence on LET (Fig. 3a), the (Z_eff_/β)^2^-dependent SSB yields for different particle types are better aligned, in particular for low energies, i.e. high (Z_eff_/β)^2^. The deviation between our results and MCDS predictions is predominantly a 10% higher SSB yield by the MCDS for high-energy ions. In the yields of DSB (Fig. 4b), only helium deviates from the unique trend seen in the present results for the other particle types, which saturate above (Z_eff_/β)^2^ ~2000. The MCDS calculations predict systematically higher DSB yields (black dotted line) than the present PARTRAC results. Especially at low energies (i.e. high (Z_eff_/β)^2^ values), these differences likely arise mainly from the different irradiation setup: First, PARTRAC does not assume electronic equilibrium conditions, but MCDS does. More importantly, a realistic slowing-down scenario is simulated in PARTRAC, whereas the shown MCDS results refer to simulations in a short DNA segment scaled to the whole cell nucleus as if uniformly irradiated with slow ions with a constant energy over the whole nucleus^17^. Consequently, in MCDS the (Z_eff_/β)^2^ values can be directly determined from the particle energy, while in PARTRAC they are determined from mean energies within the nucleus. In both approaches, low-energy results suffer from large systematic uncertainties.

The present results on (Z_eff_/β)^2^-dependent yields for DSB sites (Fig. 4c) show a peaked shape similar to the LET-related results (Fig. 3c); compared to the LET-dependent results, the agreement of hydrogen and helium between each other and with the other particle types is improved whereas the uniqueness for the four heavier particles is reduced when plotted in terms of (Z_eff_/β)^2^. The (Z_eff_/β)^2^-dependent yields for DSB-clusters (Fig. 4d) again show over a wide range of (Z_eff_/β)^2^-values, in particular below 5,000, improved uniqueness when all particles types are considered.

### LET-dependent DNA fragmentation

**Figure 5:**
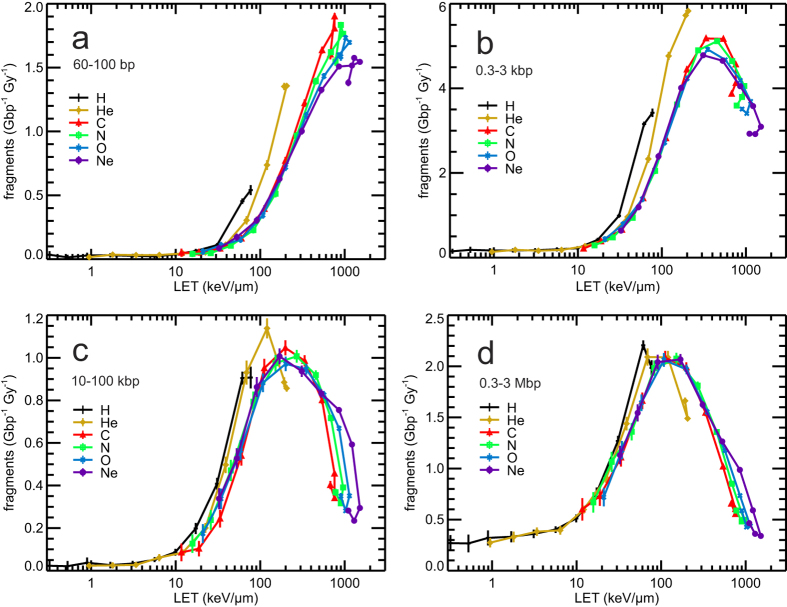
Simulation results on the yields of DNA fragments by single ion tracks. Simulation results are shown by symbols, connected by lines to guide the eyes. Error bars denote two standard errors of the mean of the performed simulations. The fragment size intervals (panel a: 60–100 bp; panel b: 0.3–3 kbp; panel c: 10–100 kbp; panel d: 0.3–3 Mbp) correspond to fragments formed when an ion track induces a pair of DSB sites within a nucleosome, chromatin fiber, a chromatin fiber loop, and a chromatin domain (or a giant loop), respectively.

While DSB clusters provide a measure of local complexity of DNA damage on nanometre scale, also the distribution of DNA damage on micrometre scale has been implicated in biological effects of ionizing radiation^54^,^55^. Therefore, for the studied irradiations, the yields of DNA fragments have been scored in widely varied size intervals: 60–100 bp (nucleosome); 0.3–3 kbp (chromatin fiber); 10–100 kbp (chromatin fiber loop); and 0.3–3 Mbp (spherical chromatin domain or giant loop) which is considered to be of particular importance within the LEM model^8^. The simulation results are presented in Fig. 5a–d in dependence on LET; as a function of (Z_eff_/β)^2^ the data are shown in Supplementary Fig. S7. The yields of the shortest fragments (Fig. 5a) closely resemble the local DNA damage complexity presented in terms of DSB clusters in Fig. 3d: A very few short fragments are induced at low LET, and their yields peak at ~500 keV/μm, reaching about 30-fold higher yields than at low LET; helium and in particular hydrogen ions are, at the same LET, more effective than heavier ions. With increasing fragment size (Fig. 5b–d), the peak shifts towards lower LET values, and its height relative to the low-LET yields gets lower.

### Local analysis of DNA strand breakage by low-energy ions

**Figure 6:**
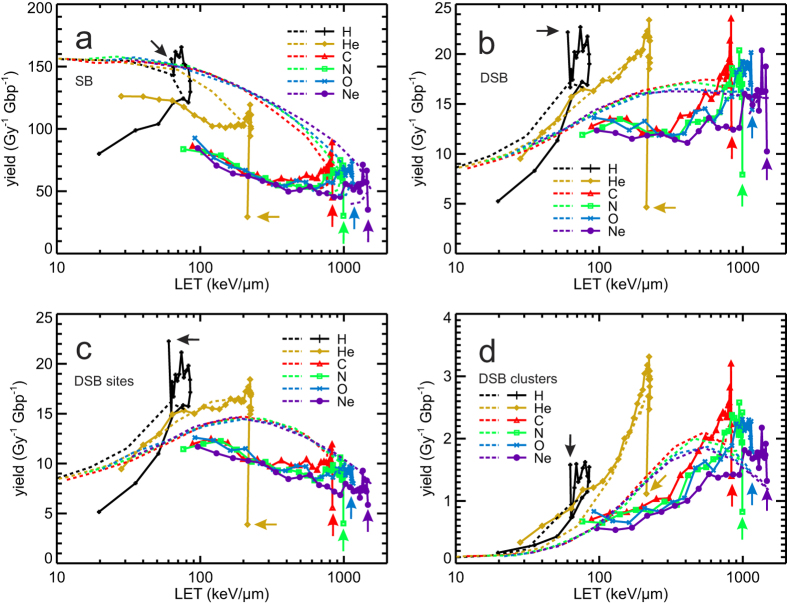
Simulated DNA damage induced in slabs of 200 nm thickness by ions with 0.25 MeV/u initial energy in dependence on the LET within that slab (Fig. 2b). Symbols connected by solid lines to guide the eye refer to subsequent slabs; dotted lines show the total damage yields in dependence on the nucleus-averaged LET (presented in Fig. 3b–d). Panel a: SB yields from direct and indirect effects. Panel b: DSB yields, in which isolated ones as well as individual DSB in DSB clusters are scored. Panel c: Yields of DSB sites, which comprise isolated DSB and DSB clusters; a DSB cluster is scored as one DSB site. Panel d: Yields of DSB clusters; a cluster includes two or more DSB within not more than 25 bp distance. The LET and the yields of DNA damage in the first slab are indicated by arrows; when the ion slows down, the damage yields proceed along the solid lines.

Finally, to study the variability in DNA damage induction within the nucleus irradiated by low-energy ions whose energy and hence stopping power largely vary along their traversal through the cell nucleus, the model results on SB, DSB, DSB sites and DSB clusters are shown in Fig. 6a–d in dependence on LET evaluated locally in 200 nm slabs of the penetration depth (i.e. LET presented in Fig. 2b). The results obtained for 0.25 MeV/u ions (symbols connected by solid lines) are also compared to the yields in dependence on the nucleus-averaged LET (dotted lines, cut-out from Fig. 3a–d). For clarity, the first slabs have been depicted by arrows; note that these initial values have large uncertainty due to the very low amount of DNA in these slabs. For 0.25 MeV hydrogen ions, for instance, the nucleus-averaged LET is about 80 keV/μm and the SB yield in the whole nucleus amounts to ~120 Gy^−1^ Gbp^−1^. The quantification in terms of depth-dependent LET in Fig. 6a provides a detailed picture (black points and line): In the first slab (black arrow) the LET is about 60 keV/μm and increases up to about 85 keV/μm in the 13^th^ slab (at 2.5 μm depth); then it starts to decrease with further increasing depth. The SB yields peak at ~165 Gy^−1^ Gbp^−1^ in the 7^th^ slab, decrease to ~130 Gy^−1^ Gbp^−1^ in the region of the maximal LET, and then go further down with decreasing LET at increasing depth. The strand breakage by very low-energy ions (i.e. on the distal branch of the single-particle Bragg peak; symbols connected by solid lines) is lower, typically by factor of about two, than that by ions that possess the same LET at higher energy (on the proximal branch of the Bragg peak; dotted lines). This is an example of overkill due to thin-down in radiobiology^56^: At track ends (i.e. at very low energies), compared to higher energies, the same energy per unit path length (the same LET) is deposited within a track that is much narrower. Consequently, unnecessarily too much energy is delivered to the DNA (in the case of direct effects) or radicals are removed by mutual reactions and/or attack the DNA often on a place hit already (for indirect effects). In Fig. 6b–d, the corresponding results are presented for DSB, DSB sites, and DSB clusters. For He only minor differences are observed in the efficiencies to induce DSB between lower- and higher-energy ions with the same LET. For heavier ions, slow particles with LET below ~70% of the maximal values tend to induce considerably less DNA damage than higher-energy ions at the same LET (Fig. 6b–d). With decreasing LET as the particles further slow down, local damage yields remain constant for DSB (Fig. 6b), increase slightly for DSB sites (Fig. 6c), and decrease about linearly for DSB clusters (Fig. 6d). Note that the DNA damage characteristics are presented in Fig. 6 only above certain low-energy and corresponding low-LET limits, since for even lower energies the nuclear processes excluded from the present analysis actually dominate over electronic ones (see Supplementary Information).

Alternative presentations of DNA damage yields are provided in Supplementary Information, including yields per cell or per track, plotted in dependence on LET, initial energy or (Z_eff_/β)^2^, or in terms of RBE. Results obtained in a complementary study for ellipsoidal human fibroblast cell nuclei are also presented in Supplementary Information.

## Discussion

A systematic evaluation of DNA damage induction by light ions has been performed, for energies from 256 MeV/u down to essentially full stopping, thus covering the range of interest in ion radiotherapy. The quantity and quality of the induced DNA damage have been assessed, in terms of DNA strand breakage, yields of DSB, DSB sites, DSB clusters and their multiplicity, and DNA fragmentation over size intervals that represent break pairs in key chromatin structures. A detailed mechanistic model of the underlying processes has been used, which includes event-by-event modelling of radiation track structures, the formation of reactive species, their diffusion, mutual reactions and attacks to DNA, and scoring the resulting damage.

To simulate the underlying track structures, a phenomenological scaling procedure has been used that enables one to derive ion cross sections and stopping powers from those of hydrogen as a generalization of the well-known Barkas formula. At low energies, the denominator in Eq. (1), namely the effective charge of hydrogen squared, ensures self-consistence making the procedure applicable to hydrogen too. From intermediate energies on, the refined scaling agrees practically with the original Barkas scheme. At high energies, the scaling reduces to that of the atomic number squared. The refined scaling provides solid results for light ions such as lithium or carbon, and works surprisingly well even for ions as heavy as neon (Supplementary Information). The procedure extends the applicability of the effective charge concept and the possibility to perform track-structure studies down to energies as low as 10 keV/u.

Around this low energy limit, interactions with nuclei of the atoms of the traversed medium become important. According to SRIM^25^ calculations, nuclear stopping power is comparable to the electronic one e.g. at energies around 1.5 keV/u for carbon ions; at further decreasing energies, the electronic stopping power continues to decrease but is compensated for by the nuclear stopping power that increases, so that the total stopping power is approximately constant over a wide low-energy range. The present approach does not include any nuclear interactions. As a rough approximation, it might have been extended to even lower energies by assuming constant cross sections and stopping powers for energies below a few keV/u; ion-specific values are listed in Supplementary Information. However, the present work has not been extended to this speculative region but used 10 keV/u as the low-energy limit on the performed track structure-based studies.

Since the present study has included electronic processes only, it has also not accounted for fragmentation of the projectile ions. Such reactions are known to play important roles in ion radiotherapy; they lead to the presence of tails beyond the Bragg peaks of light ions^1^. Nevertheless, although e.g. about 40% of 250 MeV/u carbon ions undergo a fragmentation process over their range of 13 cm, it is the primary ions that contribute dominantly to the beam’s biological effectiveness^57^. DNA damage induction by a fragmenting carbon beam has been previously addressed with preliminary PARTRAC calculations^58^, but full approaches coupling ion transport and track structure calculations should be preferred. The practical relevance of the present results to ion radiotherapy would definitely be increased when complemented by a realistic Bragg peak model accounting for energy-loss straggling, lateral scattering, and in particular nuclear reactions^59^.

The present results have been obtained by track-structure studies that have used a unique setup for high- and low-energy ions, with a source directly touching cell nuclei. Details of the irradiation geometry such as the cell and nucleus shapes or thickness of the cytoplasm significantly affect the resulting DNA damage, especially for very low-energy (short-range) particles. The reported results are most directly relevant to experiments in which the beam has to traverse only a minimal thickness of material before reaching the cell nuclei.

Especially for high-energy ions, using this setup means that electronic equilibrium conditions have not been established yet. Indeed, for high-energy ions, their LET values derived from the energy deposited within the nucleus are by up to 20% lower than the ions’ stopping powers (Fig. 2a). To address the role of electronic equilibrium in more detail, test simulations have been performed with the source of high-energy ions placed up to 10 mm away from the nucleus and with ion energies correspondingly enhanced so that they hit the nucleus with the same energy as in the reported simulations. The results have included the contribution of high-energy secondary electrons, resulting in doses and LET values up to 25% higher than the ones reported in this paper. The secondary electrons possess rather low LET and hence according to Fig. 3 induce DNA damage slightly less efficiently than the primary high-energy ion (where the small contribution of secondary electrons is included). As a consequence, under the conditions of electronic equilibrium the DNA damage yields per unit dose are slightly lower than the values reported here (differences are within a few percent), and correspond to an LET value which is higher by up to 25%.

For low-energy ions, electronic equilibrium is established over small transport lengths, and the LET and stopping power values are almost identical. At very low energies, however, stopping power varies significantly along the particle traversal through the cell nucleus. Rather than considering track-average LET, it is advantageous to define LET locally, e.g. over slabs within the nucleus, as done here on a grid of 0.2 μm (Fig. 2b).

In this work the induction of DNA damage has been simulated, and SB, DSB, DSB sites, DSB clusters and their multiplicities have been scored. Generally, with increasing LET, the SB tend to decrease. However, individual SB aggregate as DSB, whose yields increase with LET up to saturation above about 300 keV/μm. The formation of DSB sites, which include isolated as well as clustered DSB, increases up to 200 keV/μm LET, where the increasing formation of DSB clusters is counterbalanced by a decrease in isolated DSB. Then the formation of DSB clusters increases further but is overweighed by the strong decrease in isolated DSB. Finally above 500 keV/μm also the number of DSB clusters decreases with increasing LET, whereas their complexity in terms of the number of included DSB (DSB multiplicity) continues to rise; even the clusters tend to merge into more complex ones. The difference between low- and high-LET radiations is especially pronounced for DSB clusters and for the shortest DNA fragments; this reflects the high local (nm-scale) complexity of DNA damage induced by high-LET radiation.

The LET-dependent yields of DSB sites (Fig. 3c) and of Mbp-sized DNA fragments (Fig. 5d) exhibit maxima at LET around 100–200 keV/μm which are 2–8 times higher than the low-LET yields, and yields are decreasing when LET further increases beyond the maximally effective one. Such behaviour is the one that is typically seen in LET-dependent cell killing, across numerous cell lines^51^. In several studies, when such a correlation has been found for a class of DNA damage, it has been used to indicate that the given lesions may represent the initial damage that finally leads to cell killing^60^,^61^,^62^. Following this linking, the present results would indicate the biological importance of DSB sites, on the nanometre scale, and/or of ~1 Mbp fragments i.e. DSB pairs on a giant loop or within a chromatin domain, on the micrometre scale. Indeed, based on novel experiments with sub-micrometre focusing of ion beams, both scales have been implicated in the induction of chromosome aberrations and cell killing^63^,^64^. However, additional studies on the processes that link the initial damage with the induction of chromosome aberrations and cell killing are needed to elucidate whether the mentioned candidate initial lesions do represent real causal precursors of cell inactivation, or whether they merely correlate with this endpoint.

The present results show that LET defined by the energy deposited to the cell nucleus is a useful descriptor of the radiation quality. In particular, the DNA damage clustering on nanometre scales can well be attributed to regions of high LET-values. However, when hydrogen and helium particles approach their maximum LET, their effectiveness gets higher than that of heavier particles at the same LET. Moreover, the differential analysis of the damage due to ions of very low energy has indicated that on the distal branch of the single-particle Bragg peak the LET dependent DNA damage tends to be lower than on its proximal branch. Describing radiation quality in terms of effective charge squared over the velocity squared has been often proposed in the literature^49^. A principle advantage of this parameter is that it provides a functional dependence on radiation quality whereas LET values below its maximum may refer to two different radiation qualities on the proximal and distal sides of the single-particle Bragg peak. Considering DNA damage induction in dependence on (Z_eff_/β)^2^ largely removes the deviation of hydrogen and helium results from those for heavier particles; so far (Z_eff_/β)^2^ is a descriptor of radiation quality superior to LET. On the other hand, damage yields per track and among the heavier particle types also per dose are described by LET more uniquely than by (Z_eff_/β)^2^. Moreover, unlike (Z_eff_/β)^2^, LET has a clear physical interpretation, it is easy to estimate from track structure simulations, and for a given fluence it is directly proportional to dose. Finally, based on the standard Barkas formula in Eq. (2), (Z_eff_/β)^2^ approaches with decreasing particle energy an upper limit of 15,625 Z^2/3^; this leads to extremely steep gradients at very low energies^17^.

The LET determined on a slab-by-slab basis provides a differential, local picture of the efficiency for DNA damage induction (Fig. 6). Such a local description is especially important for ion energies lower than the ones at which the maximal stopping power is achieved (e.g. 0.25 MeV/u for carbon or 0.40 MeV/u for neon ions based on the ICRU data^24^). When the cell nucleus-averaged LET decreases with decreasing initial energy, hooks appear in DNA damaging effectiveness when plotted against this LET value (Fig. 3), and a differential analysis provides insight into the depth-dependent DNA damage yields (Fig. 6). Plotted as a function of (Z_eff_/β)^2^ the yields per Gy and Gbp for the four heavier particle types would rise at the high-(Z_eff_/β)^2^ ends of the lines ((Z_eff_/β)^2^ values between 25,000 and 60,000) to about 50–60 SSB (Fig. 4a), i.e. twice the value of the integral result, and decrease to 12–13 DSB (Fig. 4b), enhancing the drop already visible in the integral results.

A few track-structure based studies on ion induced DNA damage have been published so far. Nikjoo *et al*. investigated in particular the local complexity of DNA lesions after proton (0.3–4 MeV) and alpha particle (0.5–2.5 MeV/u) irradiation^65^. The results showed no major difference between protons and alpha particles at the same LET; for the LET region between 9 and 170 keV/μm, the merged data set of both particles predicts a slight (12%) decrease for SSB induction and a 2.2-fold increase for DSB induction. According to the present work, a similar increase of DSB yields is observed, whereas the decrease for SSB induction is higher (about 40%). A recent Monte Carlo simulation including proton and alpha-particle induced DNA strand breaks^66^ resulted in quite similar DSB yields within the studied LET range up to 39 and 163 keV/μm for protons and alpha particles, respectively. Trends in SSB yields due to direct and indirect effects were also comparable, with ~10% higher absolute values, whereas the reported ~5% higher yields for alphas due to indirect effects has not been obtained in our study. Another recent modelling study on ion induced direct DNA damage^67^ revealed for protons with increasing LET (up to 30 keV/μm) a linear decrease of SSB yields and linear increase of DSB yields; for a constant LET value of 24 keV/μm, the SSB yields increased and the DSB yields decreased for He, Li and C with increasing atomic number. The present results on direct DSB yields show the same trends at about 20% lower yields, whereas the about 20% lower direct SSB yields exhibit no clear trend with atomic number and no linear decrease with increasing LET.

Our earlier detailed study on proton induced DNA damage^68^ was based on a different setup, a simpler DNA model and adopting a nuclear density of 1.06 g/cm^3^; it resulted in somewhat higher absolute yields whereas the LET dependence of SB and DSB including the contributions of direct and indirect effects were the same. The investigation for helium^31^ showed up to the highest possible LET value an increasing trend with a maximum RBE for DSB induction (RBE_DSB_) of about 2.4 and a maximum induction of DNA fragments over a wide range (1–1000 kbp) around 100 keV/μm LET. Our earlier simulation of DNA damage patterns induced by boron, sulphur and the particle types considered here at the energy of 6.25 MeV/u already revealed saturation behaviour for DSB induction above 300 keV/μm LET, and for helium and hydrogen higher yields than for heavier particles at the same LET^61^. The experimental results for nitrogen^53^ included in Fig. 3f and Supplementary Fig. S9 have been investigated in great detail with PARTRAC^69^; this resulted in an underlying constant RBE_DSB_ of 2.3 between 125 and 225 keV/μm LET. In the present work the RBE for DSB sites culminates in this interval at a slightly lower RBE of 2.15 whereas the RBE_DSB_ is still slightly rising in dependence on LET at about the same value.

Further investigations in this context have been published in the framework of the semi-phenomenological MCDS algorithm^15^,^16^,^17^. In most MCDS publications, DNA damage patterns simulated in a short DNA segment have been scaled to the whole cell nucleus. For a realistic assessment of the effects induced by low-energy ions, MCDS simulations would have to be performed in sub-volumes of the nucleus that are exposed to approximately uniform irradiation. However, such an approach exceeds the scope of this paper; for the sake of simplicity, previously published MCDS results have been used in this work in comparisons with the novel PARTRAC results. Nevertheless, the present results may inform further development of the MCDS tool: PARTRAC simulations predict variations in DSB induction by different ion types (Fig. 4, Supplementary Fig. S8) that are not considered in MCDS. Further, although the simulations for very low-energy ions suffer from large uncertainties in both PARTRAC and MCDS, the results (Fig. 6) predict that the induction of DNA damage, in particular of DSB and their complexity in the distal part of the single-particle Bragg peak is significantly lower than in its proximal part, while the MCDS leads to a saturation at low energies only^17^.

Taken together, in this work, the quantity and quality of DNA damage upon irradiation with ions over a wide range of energies relevant to radiation therapy have been assessed systematically on a detailed mechanistic basis. The results have been based on detailed event-by-event track-structure simulations, thoroughly benchmarked against experimental data on interaction physics, radiation chemistry, and biophysics and biochemistry of DNA damage induction. In particular, it has been quantified how the complexity of DNA damage on the nanometre scale and its clustering on the micrometre scale vary with increasing LET, and indications have been obtained that both scales might be related to the induction of biological effects such as chromosome aberrations or cell killing. Results analogous to those presented here have also been recently employed to demonstrate the origin of the energy dependence of neutron RBE^29^,^30^. The comprehensive PARTRAC predictions of DNA damage as a function of charged particle LET in the cell nucleus constitute a rich database, which can be queried for different applications. In particular, the results help improve mechanistic understanding of the high biological effectiveness of ion radiotherapy and may inform radiation protection in space research.

## Additional Information

**How to cite this article:** Friedland, W. *et al*. Comprehensive track-structure based evaluation of DNA damage by light ions from radiotherapy-relevant energies down to stopping. *Sci. Rep.* **7**, 45161; doi: 10.1038/srep45161 (2017).

**Publisher's note:** Springer Nature remains neutral with regard to jurisdictional claims in published maps and institutional affiliations.

## Supplementary Material

Supplementary Information
