# Supplementary material for: Comprehensive track-structure based evaluation of DNA damage by light ions from radiotherapy-relevant energies down to stopping
Source: Sci Rep. 2017 Mar 27;7:45161. doi: 10.1038/srep45161 (PMC5366876; doi:10.1038/srep45161)
Supplement: Supplementary Information [file srep45161-s1.pdf]

Comprehensive track-structure based evaluation of DNA damage by light ions from radiotherapy-relevant energies down to stopping

W. Friedland[1,*], E. Schmitt[1], P. Kundrát[1], M. Dingfelder[2], G. Baiocco[3], S. Barbieri[3] and A. Ottolenghi[3]

[1]Institute of Radiation Protection, Department of Radiation Sciences, Helmholtz Zentrum München – German Research Center for Environmental Health (GmbH), Neuherberg, Germany

[2]Department of Physics, East Carolina University, Greenville, NC, USA

[3]Department of Physics, University of Pavia, Pavia, Italy

*Corresponding author: friedland@helmholtz-muenchen.de

Supplementary Information

Calculation of cross sections for particles with atomic number Z > 2

In the determination of cross sections for particles with atomic number Z > 2, the Barkas scaling term[1] for the effective charge has been replaced by its relation to the Barkas term for Z=1:

$$\sigma_Z = \sigma_H\, Z^2(1 - \exp(-125(v/c)\, Z^{-2/3}))^2 \,/\, (1 - \exp(-125v/c))^2. \qquad \text{(S1)}$$

The cross section for hydrogen σ_H can be separated into a fraction due to proton interactions, σ_1, and a fraction due to neutral hydrogen interactions, σ_0:

$$\sigma_H = f_+\, \sigma_1 + (1 - f_+)\, \sigma_0\,, \qquad \text{(S2)}$$

where f_+ is the energy-dependent fraction of protons during the slowing-down process.

In a first approximation the energy-dependent fraction of proton states during slowing-down was estimated by the effective charge term for Z=1 according to the Barkas formula as

$$Z_{\mathrm{eff,H}} = 1 - \exp(-125v/c)\,. \qquad \text{(S3)}$$

This estimate resulted in energy dependent stopping powers and ranges for carbon which have been found in reasonable agreement with ICRU data[2], SRIM calculations[3] and recent measurements[4]. However, a comparison of this approach with a usual Monte Carlo slowing-down calculation for hydrogen has shown deviations in the energy-dependent charged fractions, indicating that the underlying approach was not fully self-consistent. Indeed, the fractions of charged states f_+ and neutral states f_0 can be derived[5,6] due to their dynamic equilibrium from the cross sections for electron stripping of neutral hydrogen σ_{01} and electron pick-up by protons σ_{10}

$$f_+ = \sigma_{01} \,/(\sigma_{01} + \sigma_{10}), \qquad \text{(S4)}$$

$$f_0 = 1 - f_+ = \sigma_{10} \,/\, (\sigma_{01} + \sigma_{10}). \qquad \text{(S5)}$$

For application in the track structure code, however, the primary quantity is the mean free path λ_Z, i.e., the inverse of the cross section multiplied by the given target density N. The scaling law for λ_Z is

$$\lambda_Z = (1 - \exp(-125v/c))^2\, \lambda_H \,/\, Z_{\mathrm{eff}}^2. \qquad \text{(S6)}$$

From Eqs. (S2, S4, S5) one gets

$$\lambda_H N = 1 / \sigma_H = (\sigma_{01} + \sigma_{10}) / (\sigma_{01} \sigma_1 + \sigma_{10} \sigma_0)$$

$$= ((\sigma_{10} / \sigma_1) / \sigma_0 + (\sigma_{01} / \sigma_0) / \sigma_1) / ((\sigma_{10} / \sigma_1) + (\sigma_{01} / \sigma_0))$$

$$= p_+ (1 / \sigma_1) + p_0 (1 / \sigma_0) \quad \text{(S7)}$$

with

$$p_+ = (\sigma_{01} / \sigma_0) / ((\sigma_{10} / \sigma_1) + (\sigma_{01} / \sigma_0)) \quad \text{(S8)}$$

and

$$p_0 = 1 - p_+ = (\sigma_{10} / \sigma_1) / ((\sigma_{10} / \sigma_1) + (\sigma_{01} / \sigma_0)). \quad \text{(S9)}$$

Thus, the change from cross sections to their inverse quantities, the mean free paths, entails a transition from fractions of charge states, Eqs. (S4-S5), to fractions of charge-changing processes, Eqs. (S8-S9). The algorithm according to Eqs. (S6-S9) has been implemented in the PARTRAC code for the transport calculation of particles with $Z > 2$; differential cross sections are taken from the proton or neutral hydrogen data sets with probabilities according to Eqs. (S8-S9).

In Figure S1, various data on fractions of charged states are presented for hydrogen particles as a function of energy, including the fractions f_+ (Eq. (S4)), p_+ (Eq. (S8)) and the earlier Barkas approximation $Z_{eff,H}$ (Eq. (S3)). At low energies the fraction of charged states is rather low (e.g. less than 10% below 5 keV). However, even at such low energies the fraction of stripping processes among interactions of neutral hydrogen states is about 25%. Therefore, on average after 3 inelastic interactions of neutral hydrogen the electron is stripped, and subsequently a proton interaction will happen; with high probability (below 5 keV above 80%) this will be an electron pick-up process yielding immediate re-formation of the neutral state after just a short transport distance. Thus, the fraction p_+ of proton interactions regarding the sequence (or probability) of processes is about 20% at the low energy limit.

Characteristic for charge changing processes during hydrogen slowing-down is that the number of electron pick-up processes is always equal to or exceeds by one the number of electron stripping processes. In order to safeguard this balance between both processes in a Monte Carlo procedure, in this work they have been combined into a single process including an ionization of the liquid water medium (due to pick-up) and an emission of a secondary electron with the speed of the energetic particle in forward direction (due to stripping). The combined charge-changing processes have been associated to interactions of neutral hydrogen states. However, pick-up processes by protons must not be excluded in the particle transport. To obtain the same transport behaviour in the approximate track structure simulation, and consequently the correct stopping power, electron pick-up processes have been included as transport events without any interaction.

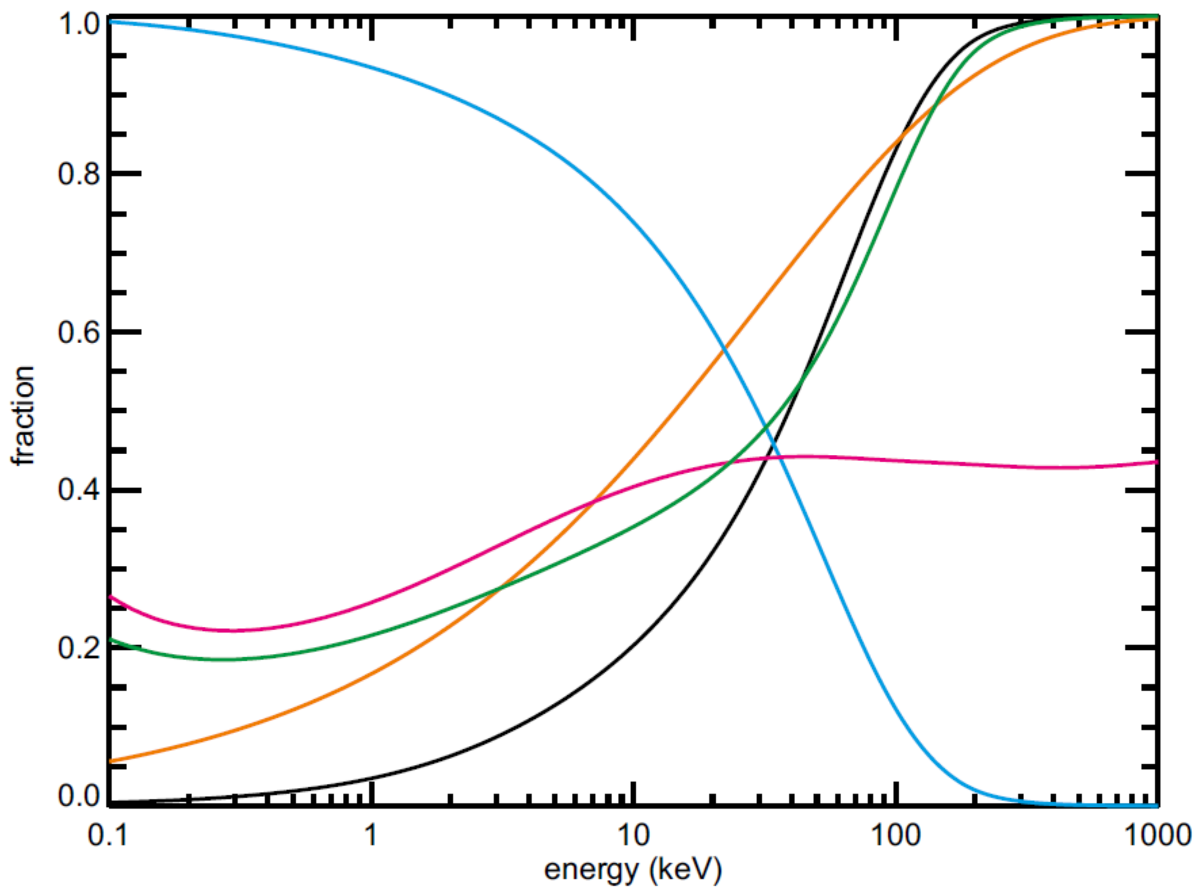

Figure S1. Fraction of processes and charge states during hydrogen slowing-down. Cyan line: fraction of electron pick-up processes among inelastic interactions of protons; magenta line: fraction of electron stripping processes among inelastic interactions of neutral hydrogen; black line: fraction of hydrogen particles in charged state (Eq. (S4)); green line: probability of charged hydrogen interaction (Eq. (S8)); orange line: Z_{eff} according to Barkas formula for hydrogen (Eq. (S3)). Data apart from Z_{eff} are based on cross sections by Dingfelder et al.[6].

Validation of cross-section scaling

To test the validity of the generalized Barkas procedure, Eq. (1), it has been applied directly to stopping powers. In Fig. S2a-d, the electronic stopping powers for He, Li, C, and Ne ions (solid red lines) obtained by scaling the ICRU data for hydrogen[7] are compared to the standard values (black points) recommended by the ICRU[2]. In Fig. S2, solid green lines depict stopping powers obtained by an analogous scaling method that starts from ICRU data for helium[7] instead of hydrogen; this procedure exactly reproduces the He data (Fig. S2a), verifying its self-consistence. The hydrogen-based scaling underestimates the maximal stopping power for helium (Fig. S2a) by about 4% and shifts its location to about 40% lower energies compared to the ICRU database (217 keV/µm at 0.1 MeV/u vs. 226 keV/µm at 0.18 MeV/u); this reflects the very different atomic structure of helium compared to that of hydrogen. Note, however, that the scaled results for helium are shown here for comparison only; they are not used in PARTRAC, since in the code there is a dedicated cross-section database for helium ions[8] that explicitly accounts for its charge states. For light ions with Z > 2 (Figs. S2b-d), the stopping powers from hydrogen-based generalized Barkas scaling (solid red lines) are in

good agreement with the ICRU data[2], but underestimate the maximum stopping powers by a few percent. The generalized scaling works much better than the original one (dashed red lines), especially at energies below ~0.5 MeV/u.

In the literature, also a three-term expression can be found for the effective charge[9],

$$Z_{eff}(v) = Z\left(1 - \exp\left(-131.6(v/c)Z^{-2/3} + 1112(v/c)^2 Z^{-4/3} - 65000(v/c)^3 Z^{-2}\right)\right). \quad (S10)$$

As illustrated for carbon ions (Fig. S2c, dash-dotted red line), this alternative effective charge formula, Eq. (S10), produces results very close to those from Eq. (2), used throughout this work.

The refined Barkas scaling works surprisingly well for ions as heavy as neon (Fig. S2d). For all studied light ions with Z > 2, scaling based on hydrogen is superior to the one that starts from helium data (solid and dashed green lines for the refined and original procedures, respectively).

The results obtained with the scaling procedure cover only electronic stopping powers. PARTRAC does not include nuclear interactions, and hence it does not assess nuclear stopping powers or fragmentation of the projectile ion in nuclear reactions. ICRU data are available on nuclear stopping powers for helium[7] (Fig. S2a, blue points), and SRIM calculations[3] do reproduce these values and provide estimates of nuclear stopping powers for all the studied ions (thin blue lines in Fig. S2). Above 100 keV/u, nuclear stopping powers are negligible, and hence they have not been plotted here. At energies below about 10 keV/u, however, nuclear processes do contribute significantly to the ions' stopping. At very low energies, nuclear stopping powers exceed the electronic ones; according to SRIM calculations, this occurs below 0.17, 0.69, 1.5, 1.6, 2.0, and 3.0 keV/u for H, He, C, N, O, and Ne ions; the corresponding values of nuclear and equally electronic stopping powers are 6, 18, 76, 91, 104 and 123 keV/µm, respectively. At about twice higher electronic stopping powers, the nuclear ones amount to about a fourth of electronic ones, i.e. nuclear processes make about 20% of the total stopping only. These values can serve as estimates of the low-energy limit of the present PARTRAC simulations.

To further validate the effective charge concept, it has been adopted in Monte Carlo simulations of the full slowing-down of hydrogen. In Fig. S3a, it is shown that this method provides results (solid red line) that are fully in agreement with usual simulations (grey dotted line) that explicitly track the charge states, i.e. distinguish between neutral hydrogen atoms and singly-charged hydrogen ions (protons) and use dedicated cross sections for these two states. PARTRAC results generally agree with the ICRU data[7] (blue squares). Around the maximal stopping power, the results of PARTRAC are by up to 6% higher than the ICRU values, and closely follow the calculations by the SRIM[3] code (black crosses). To provide a more detailed picture of the processes, contributions to the stopping power have been separately scored for ionisations by protons (green line), excitations by protons (orange line), ionisations by neutral hydrogen (yellow line), and by charge changing processes (violet line).

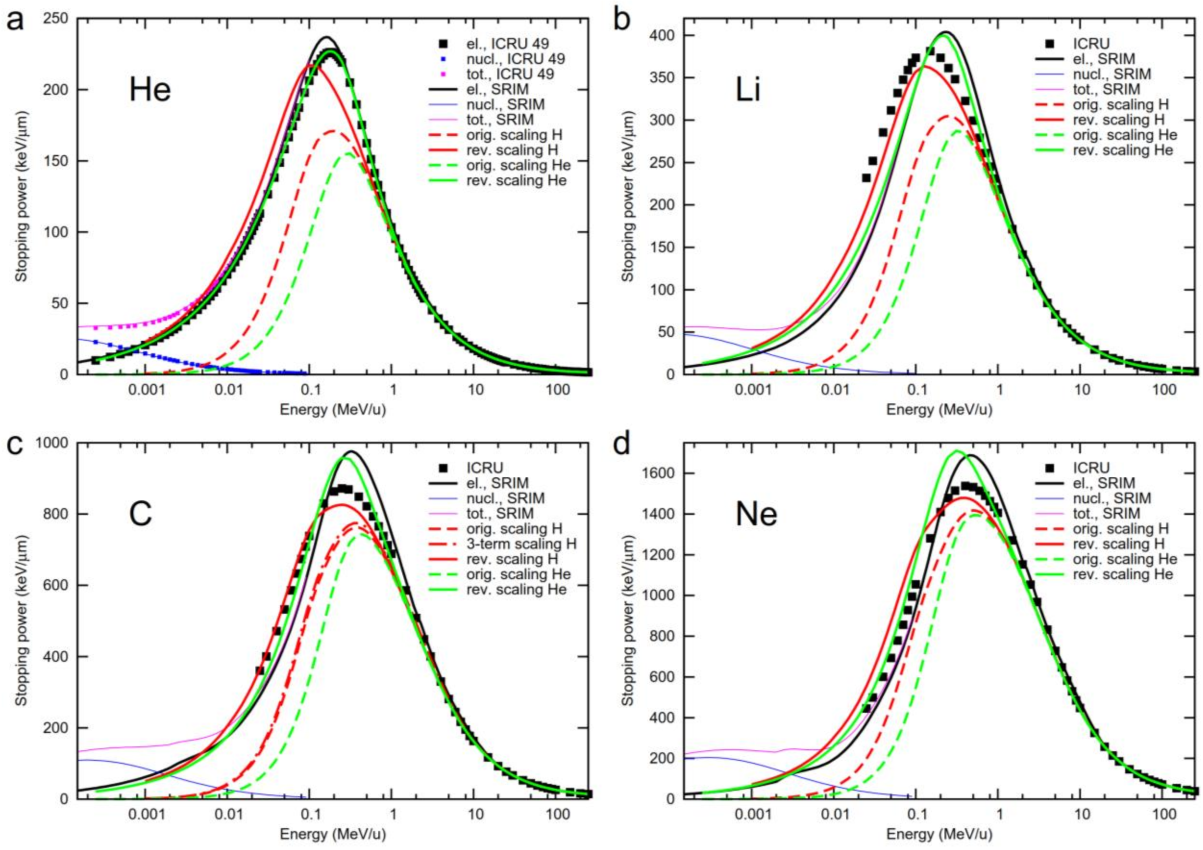

Figure S2: Energy-dependent stopping powers of ions in liquid water. Stopping powers for ions (solid red lines; panel a: He, panel b: Li, panel c: C, panel d: Ne ions) obtained by scaling according to generalized Barkas formula, Eq. (1), from the ICRU[7] hydrogen data, are compared to the ICRU[2] ion data (black symbols). The results of a similar scaling procedure based on helium instead of hydrogen ions are shown too (solid green lines). For comparison, also plotted are the results of the original Barkas scaling, Eq. (1) without the denominator, starting from hydrogen (dashed red line) or helium ions (dashed green line). For helium ions (panel a), the ICRU database contains data on nuclear and total stopping; these are shown by blue and magenta points. Also shown are electronic, nuclear and total stopping powers from SRIM[3] (black, blue and magenta lines, respectively). Since nuclear stopping is negligible above 0.1 MeV/u, it has not been included in the plots in that region. In panel c, also shown is the scaling based on an alternative formula for the effective charge, Eq. (S10).

A track-structure simulation of full stopping within the effective charge concept has been performed also for carbon ions, i.e. with interaction cross sections obtained from hydrogen data by scaling according to Eq. (1). 100 tracks of 10 MeV/u carbon ions have been simulated, and the primary particle interactions have been scored. The stopping powers from this Monte Carlo procedure (Fig. S3b, red line) are practically identical with those from directly scaling hydrogen stopping powers (Fig. S2c, solid red line). They closely reproduce the ICRU data[2] (Fig. S3b, blue squares) but underestimate the stopping power around its maximum by almost 7%; in comparison, the SRIM[3] code (Fig. S3b, black crosses) predicts the maximal stopping power to be about 10% higher and shifted to about 30% higher energies than the ICRU. In the low-energy range, the present approach perfectly agrees with the ICRU data; for energies < 25 keV/u, the low-energy limit of the ICRU dataset, the present results show trends similar to those predicted by the SRIM code.

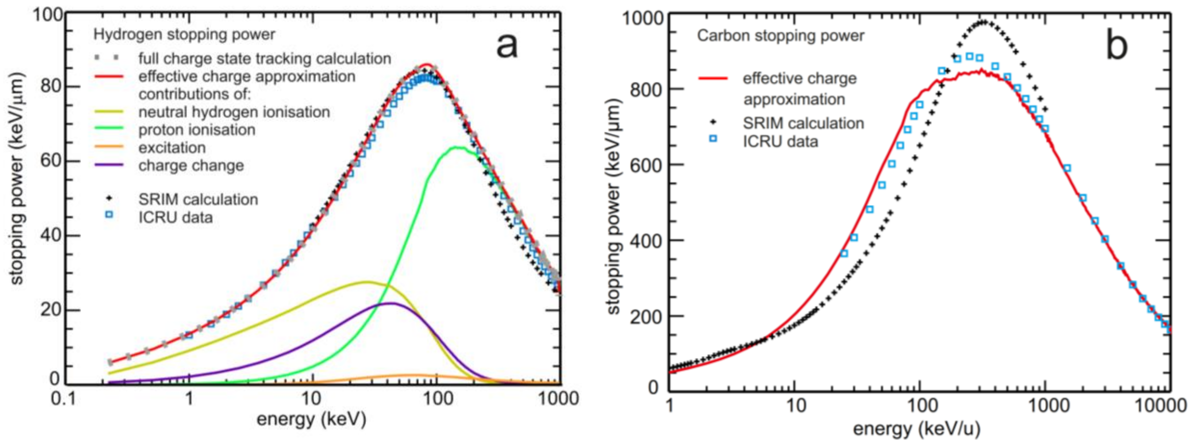

Figure S3. Monte Carlo slowing-down simulations with PARTRAC. Panel a: Slowing-down simulations that track only the effective charge of hydrogen (red line) agree with full simulations (grey dotted line) that explicitly distinguish between its charge states, neutral hydrogen atoms and singly-charged ions i.e. protons. Stopping power has been calculated from mean energy loss in adjacent 50 nm slabs. For comparison, ICRU data[7] (blue squares) and SRIM[3] calculations (black crosses) are shown. Also shown are contributions of different charge states, calculated from mean energy transfer and mean number of events in 50 nm slabs: ionisations by protons (green line), excitations by protons (orange line), ionisations by neutral hydrogen (yellow line), and the contribution by charge-changing processes (violet line). Panel b: Slowing-down simulations for carbon ions using the present effective charge concept (red line) compared to ICRU data[2] (blue squares) and SRIM calculations[3] (black crosses).

Comparison of DNA damage predictions in lymphocytes and fibroblast nuclei

Additional simulations have been performed for modelled nuclei of human fibroblasts irradiated by ^{1}H, ^{4}He, ^{12}C, ^{14}N, ^{16}O, and ^{20}Ne particles with initial energies taken from a binary grid (H: 0.0625–8 MeV; He: 0.0625–16 MeV/u; C: 0.125–32 MeV/u; N: 0.125–32MeV/u; O: 0.125–64 MeV/u, Ne: 0.25–128 MeV/u). The source of these ions has been a rectangular area of 12 µm × 20 µm size in a plane in 3 µm distance from and in parallel to the central plane of the nucleus spanned by the two larger axes of the ellipsoidal fibroblast nucleus. The source area has been divided into 15 sub-squares of 4 µm × 4 µm size. 15 ions have been started exactly parallel per simulation run, within each sub-square one from a random position, corresponding to a fluence of 0.0625 µm^{-2} (i.e. the same fluence as in the standard simulations reported in the main body of this paper). The ions were almost perpendicular to the rectangular source area; a slight random deviation from the normal direction has been introduced to avoid artefacts that would otherwise arise from the alignment of tracks with chromatin model structures. To reach sufficient statistics, 64 runs for C, N, O, and Ne ions have been performed for each energy value and energy-dependent higher numbers for H and He. Primary particles and secondary electrons were followed in a cuboid of 6 µm × 12 µm × 20 µm size surrounding the nucleus and filled with water.

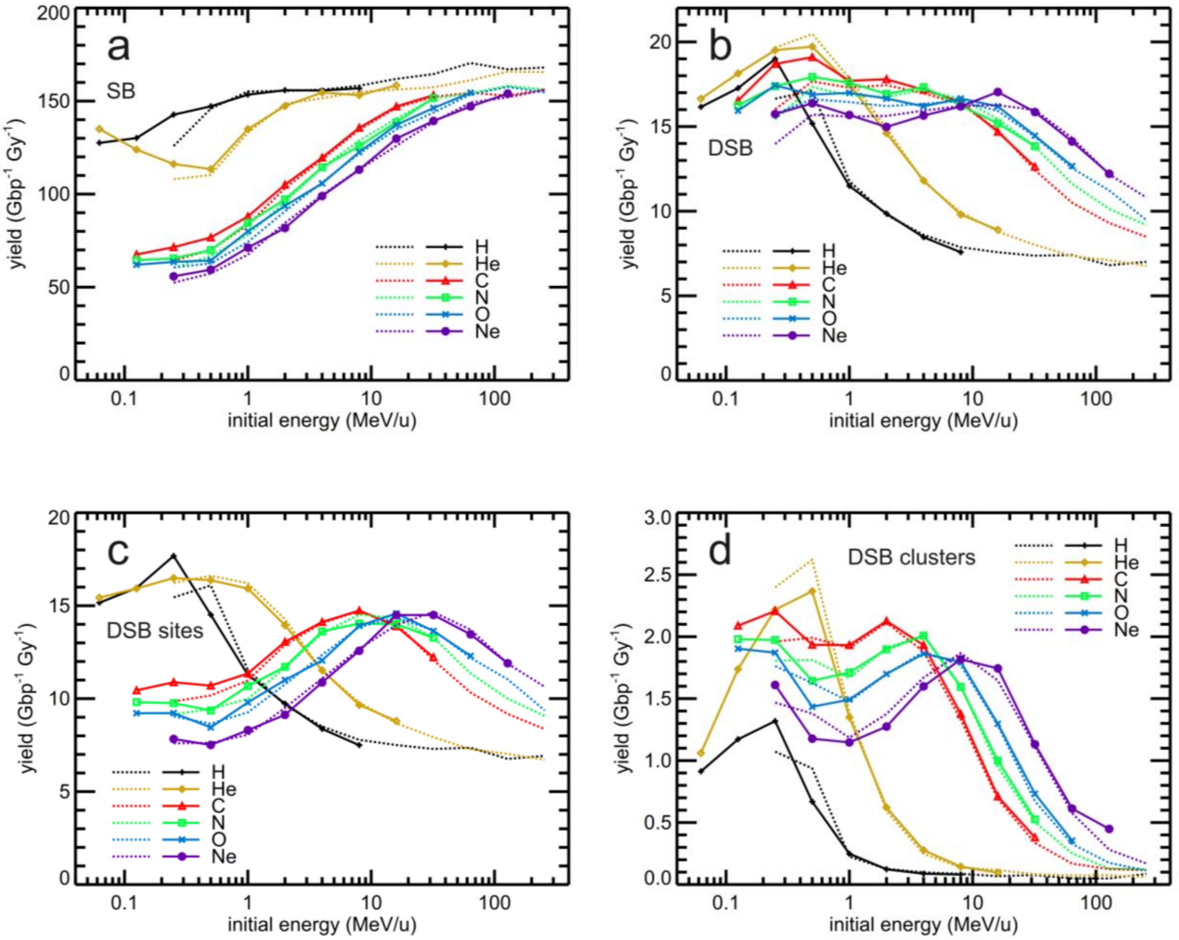

Figure S4. Ion-induced DNA damage as a function of initial energy. Simulation results for fibroblast model nucleus are shown by symbols connected by solid lines to guide the eyes. Results for lymphocyte model nucleus in dependence on LET are denoted by dotted lines (replotted from Fig. 3 but here shown in dependence on the initial energy rather than on the LET). Panel a: SB; panel b: DSB, panel c: DSB sites; panel d: DSB clusters.

Taken together, the setup for fibroblast nuclei is in several respects an alternative approach to the settings in the calculation for lymphocyte nuclei. Due to the flat shape of the nucleus and shorter transport distance, calculations with lower energies could have been performed. However, the DNA structure on scales up to 50 nm, the track structures, and the method of assessing DNA damage by overlapping the DNA model with the tracks are the same in both approaches.

To point out the effectiveness of different energies, DNA damage yields for fibroblasts are shown in Fig. S4 as a function of initial energy (symbols connected by solid lines) compared to the results for lymphocytes (dotted lines). The results for the two nuclei are in good agreement with each other; this is the case also in dependence on LET or as a function of $(Z_{eff}/\beta)^2$ (results not shown). On the other hand, the per-track yields (not shown) are lower by a factor of ~2 for fibroblasts than for lymphocytes, since the track lengths are shorter in the flat fibroblast nuclei than in the spherical lymphocytes.

Yields of DNA damage per track and lymphocyte cell in dependence on LET

Cellular DNA damage induction per track (Fig. S5) highlights its increase upon slowing down of the primary particle. For all studied endpoints the yields of DNA damage per track in lymphocyte cell in dependence on LET show continuous increase with increasing LET until the particle is stopped inside the nucleus, and the dependence on particle type is rather low as long as the particles possess energy sufficient to pass through the whole nucleus.

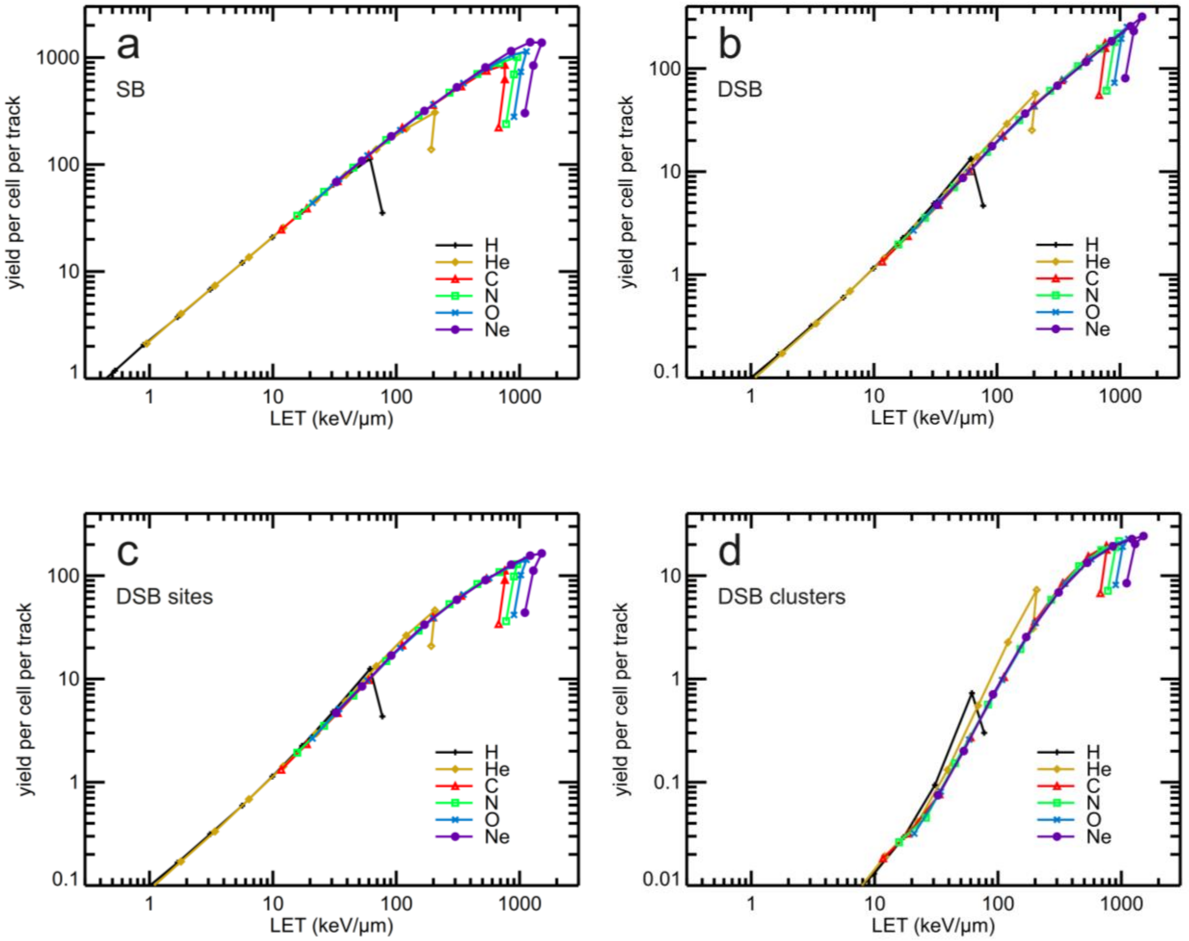

Figure S5. Ion induced DNA damage per track in lymphocyte nuclei in dependence on LET. Simulation results for lymphocyte model nucleus are shown by symbols; they are connected by solid lines to guide the eyes. Panel a: SB yields, panel b: DSB yields, panel c: yields of DSB sites, panel d: yields of DSB clusters.

Characterizing ion tracks by $(Z_{eff}/\beta)^2$

A commonly used parameter for radiation quality[10] that may provide a lower dependence on the particle type than the LET is the square of the effective charge divided by the velocity $(Z_{eff}/\beta)^2$. Similarly to the LET discussed in the main body of the paper, also $(Z_{eff}/\beta)^2$ is approximately constant for high-energy ions but for low-energy ones it varies significantly along their traversal through the

cell nucleus. To deal with this issue, values of $(Z_{eff}/\beta)^2$ have been determined in this work for the mean energy inside the nucleus, which in turn has been calculated from the mean energy loss during the passage of the particles through the nucleus. Note that these values of $(Z_{eff}/\beta)^2$ hardly represent the track-average values for low-energy ions.

For comparison of the LET and $(Z_{eff}/\beta)^2$ concepts, the values of $(Z_{eff}/\beta)^2$ and of the LET resulting from dose and fluence, Eq. (4), for these mean energy values are presented in Fig. S6. At low energies, considering the LET values in dependence on these mean energies (Fig. S6a) leads to an improved agreement with ICRU stopping power data[2,7] than if the LET is related to the initial energies (Fig. 2a). For the determination of Z_{eff}, the Barkas formula in Eq. (2) has been used for the sake of consistency with published data; the general findings would not change if the denominator in Eq. (1) were included.

The resulting $(Z_{eff}/\beta)^2$ data span a range from ~2.5 for the fastest H up to ~50,000 for the slowest Ne particles (Fig. S6b). Contrary to the (local) LET and stopping power which increase in the course of an ion's slowing down from an initial value to a maximum (single-particle Bragg peak) and then decrease in its distal part, $(Z_{eff}/\beta)^2$ steadily increases for each particle with decreasing energy. While the results plotted in dependence on LET often exhibit hooks (two distinct values at a single LET corresponding to proximal and distal parts of the single-particle Bragg peak, respectively), $(Z_{eff}/\beta)^2$ can be used as a parameter for describing the results as a mathematical (single-valued) function; the results are shown in subsequent sections.

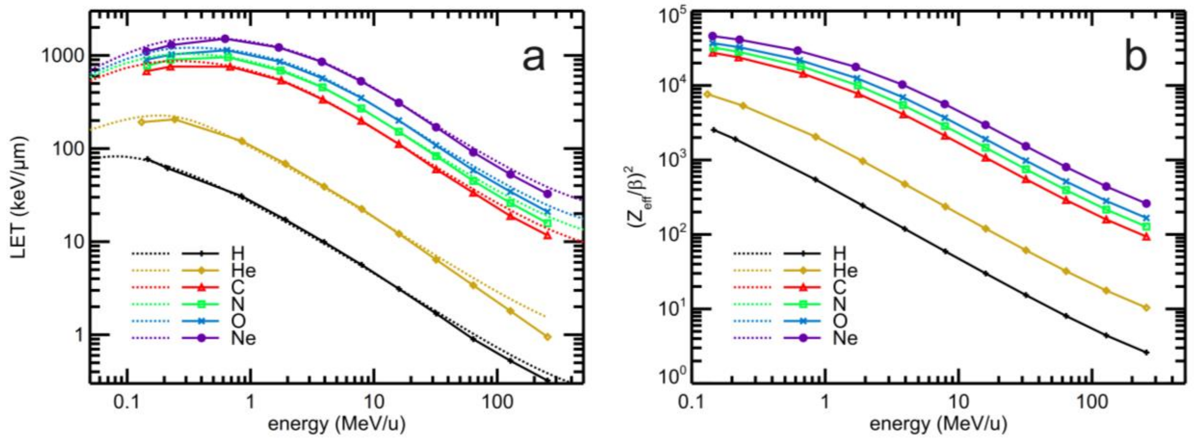

Figure S6. LET and $(Z_{eff}/\beta)^2$ as a function of the mean energy in lymphocyte nuclei. Simulation results for lymphocyte model nucleus are shown by symbols connected by solid lines to guide the eyes. Panel a: LET values as a function of the mean energy (see text) within the irradiated nucleus, compared to ICRU data[2,7] (dotted lines); panel b: $(Z_{eff}/\beta)^2$ as a function of the mean energy (see text) within the irradiated nucleus.

In Fig. S7, yields of double-stranded DNA fragments in four size intervals formed by single ion traversals are presented as a function of $(Z_{eff}/\beta)^2$. The yields for H are lower than for the other particle types, the yields for He are similar to heavier particles, and the alignment of the heavier particles is at least partly better than when plotted in dependence on LET (Fig. 4).

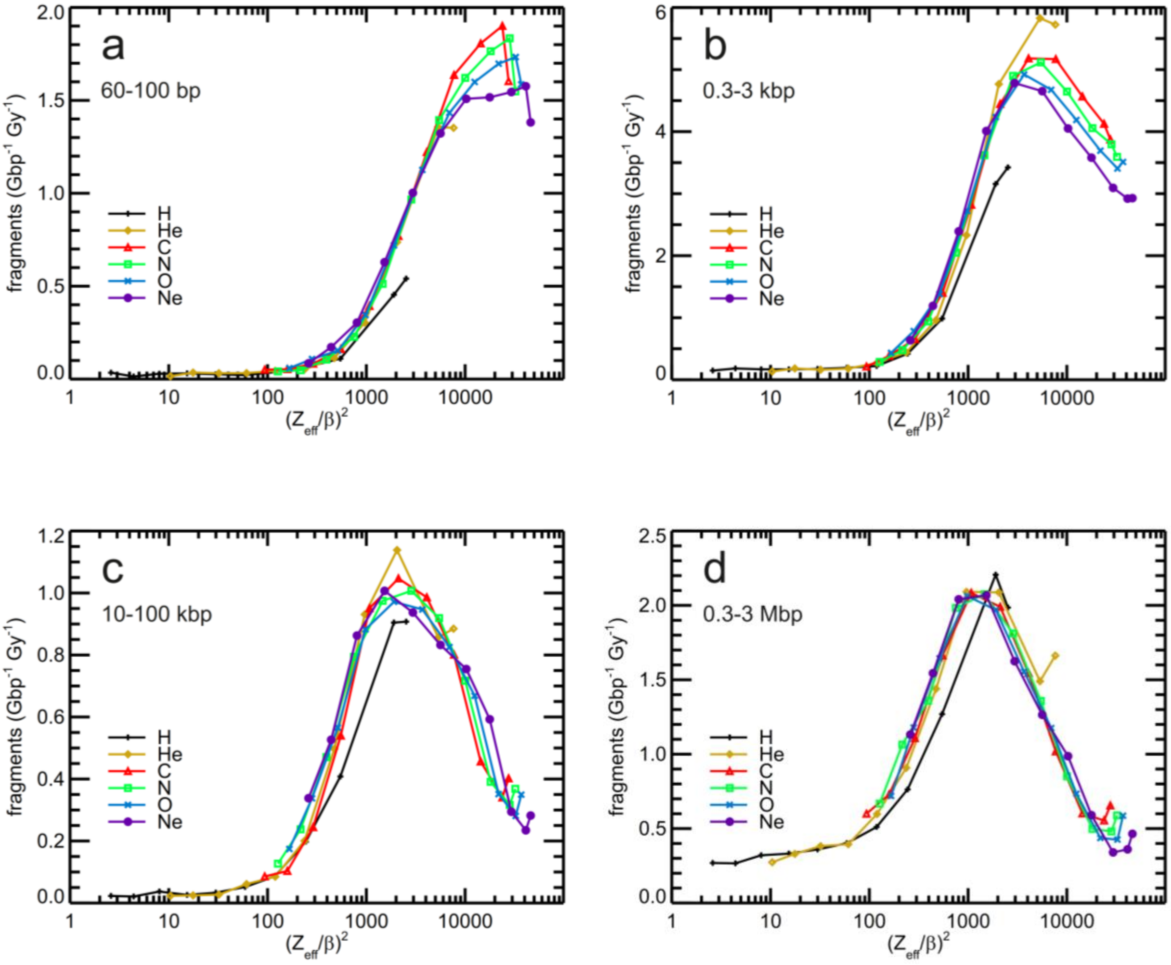

Figure S7. Double-stranded DNA fragments induced by single ion traversals in lymphocyte nuclei as a function of $(Z_{eff}/\beta)^2$. Simulation results are shown by symbols connected by lines to guide the eyes. Panel a: DNA fragments in interval 60–100 bp; panel b: DNA fragments in interval 0.3–3 kbp; panel c: DNA fragments in interval 10–100 kbp; panel d: DNA fragments in interval 0.3–3 Mbp.

When considered per ion track, the DNA damage yields in lymphocyte cell (Fig. S8) as a function of $(Z_{eff}/\beta)^2$ are for H and He significantly lower than for heavier particles; the overall good alignment seen in dependence on LET for all considered particles (Fig. S5) has been lost. Uniqueness for particles heavier than He is still present up to a $(Z_{eff}/\beta)^2$-value of about 10,000, but also reduced compared to the dependence on LET (Fig. S5).

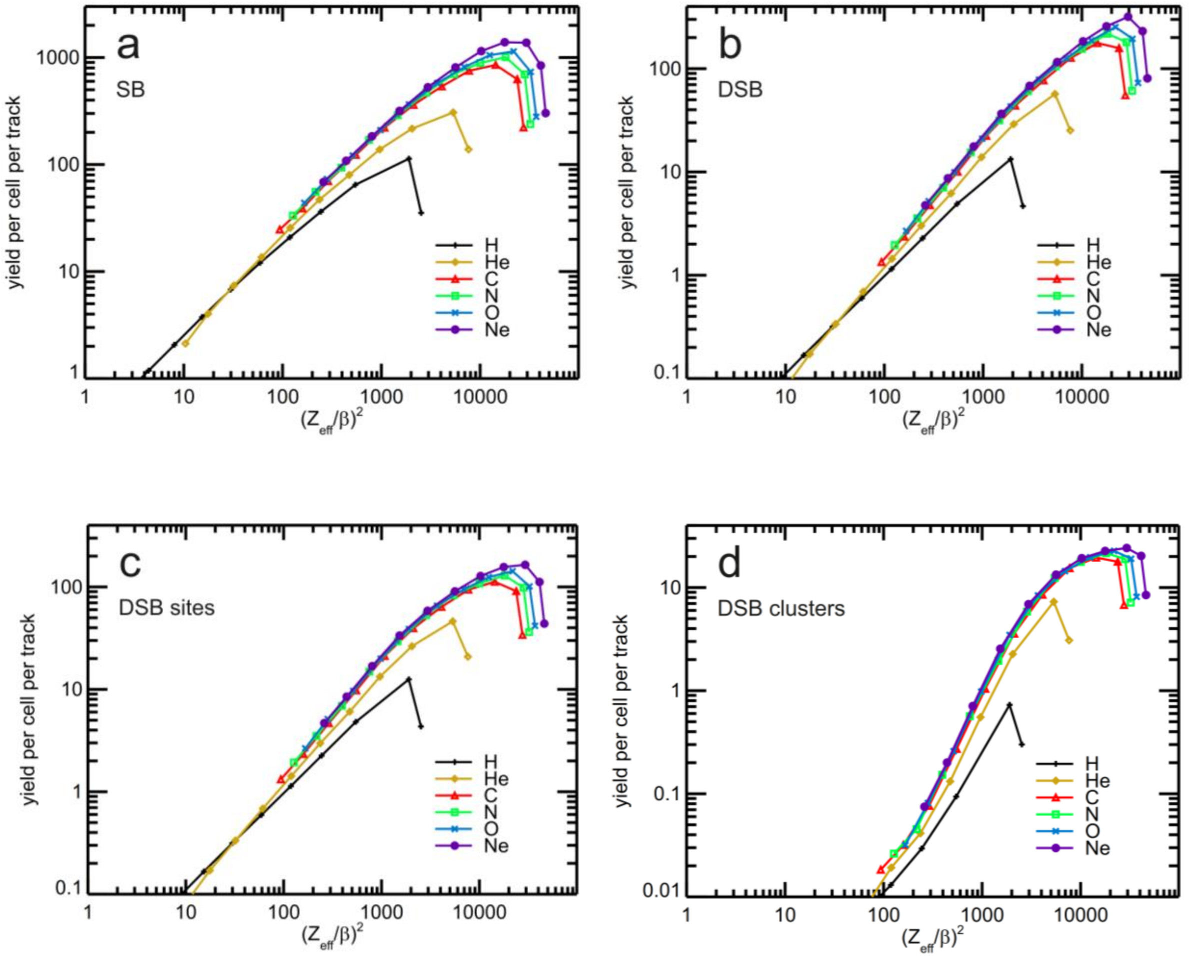

Figure S8. Ion induced DNA damage per track in lymphocyte nuclei as a function of $(Z_{eff}/\beta)^2$. Black: hydrogen, yellow: helium, red: carbon, green: nitrogen, blue: oxygen, violet: neon. Simulation results are shown by symbols connected by lines to guide the eyes. Panel a: SB yields, panel b: DSB yields, panel c: yields of DSB sites, panel d: yields of DSB clusters.

RBE in dependence on LET or $(Z_{eff}/\beta)^2$

In Fig. S9, yields of DSB, DSB sites, DSB clusters and a comparison to experimental results are presented in terms of RBE. Since no calculation for a reference photon irradiation has been performed in this work, the yields have been related to the results for 128 MeV hydrogen which turned out to yield for the endpoints considered here the lowest values of all studied radiation qualities. The RBE for DSB induction (panel a) peaks at ~3 for He but saturates for heavier particles at ~2.5, which is also the maximum value for H; the MCDS prediction[11] is slightly above the yields for He and H as long as these particles possess energy high enough to fully pass through the nucleus, but do not reflect the saturation trend for the heavier particles beginning above a $(Z_{eff}/\beta)^2$-value of ~1,000. The RBE for DSB sites (panel b) culminates at ~2.5 for H and He near their maximal LET values and at ~2.2 for the heavier particles at ~200 keV/µm LET. The RBE for DSB clusters (panel c) rises up to ~22, ~55 and ~40 for H, He and heavier particles, respectively, at the highest achievable $(Z_{eff}/\beta)^2$ (for H)

or, for the other particle types, at an overall common $(Z_{eff}/\beta)^2$-value of ~5,000. RBE values from experiments (panel d) are in excellent agreement with calculations taking into account the detectable fragment size interval according to the experimental protocol[12,13].

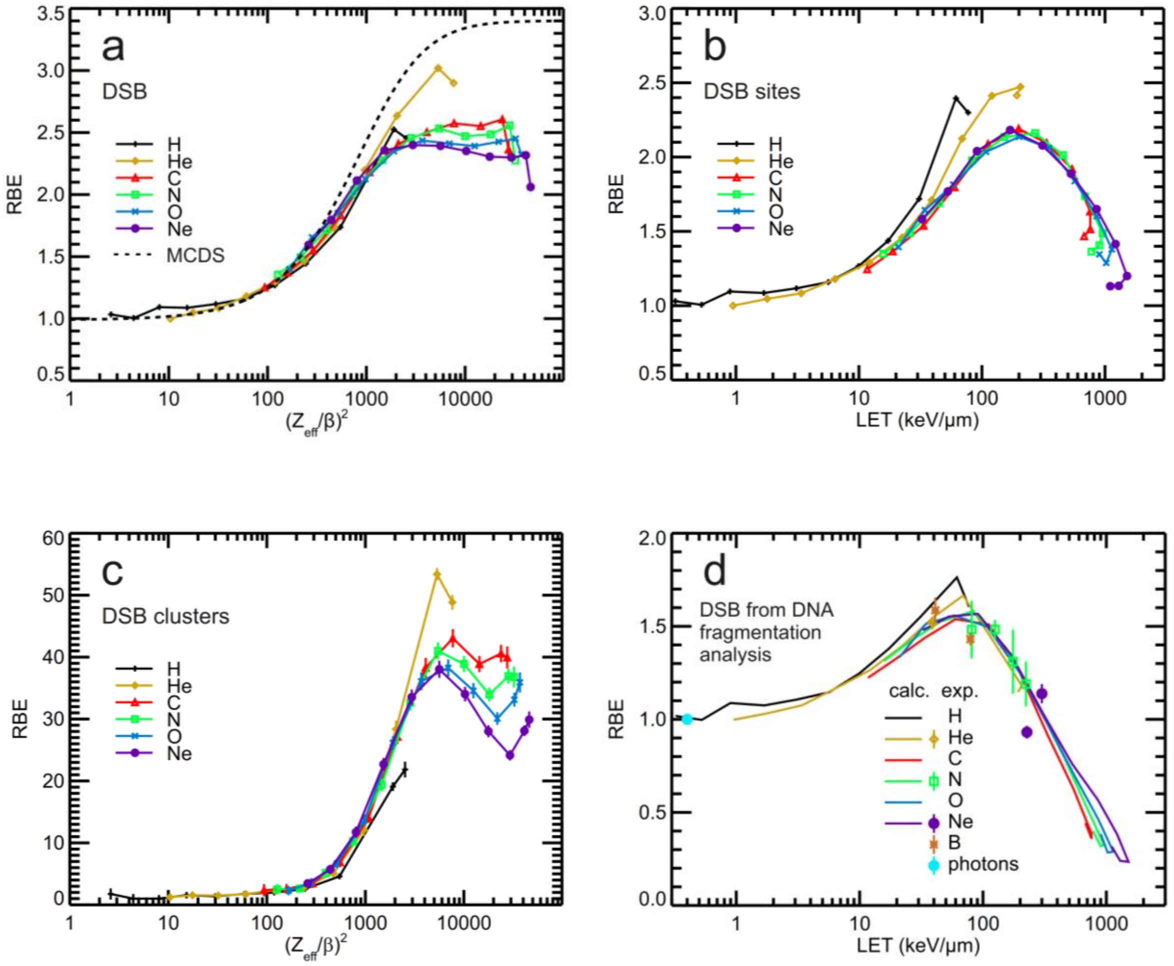

Figure S9. Relative biological effectiveness (RBE) regarding DSB, DSB sites, DSB clusters and in comparison to experimental results. Simulation results for lymphocyte model nucleus are shown in panel a-c by symbols connected by solid lines to guide the eyes, in panel d by solid lines. Panel a: RBE for DSB induction as a function of $(Z_{eff}/\beta)^2$; panel b: RBE for DSB sites in dependence on LET; panel c: RBE for DSB clusters as a function of $(Z_{eff}/\beta)^2$; panel d: RBE for DSB induction considering the detected DNA fragment size interval in comparison to experimental results[12,13] (symbols) in dependence on LET.

References

1. Barkas, H.W. *Nuclear Research Emulsions*, (Academic Press, New York, 1963).
2. Siegmund, P., Schinner, A. & Paul, H. Errata and Addenda for ICRU Report 73, Stopping of ions heavier than helium. *J ICRU* **5**, 1 (2009).
3. Ziegler, J.F., Ziegler, M.D. & Biersack, J.P. SRIM - The stopping and range of ions in matter (2010). *Nucl Instrum Meth B* **268**, 1818-1823 (2010).
4. Rahm, J.M., Baek, W.Y., Rabus, H. & Hofsass, H. Stopping power of liquid water for carbon ions in the energy range between 1 MeV and 6 MeV. *Phys Med Biol* **59**, 3683-3695 (2014).

5. Miller, J.H. & Green, A.E. Proton energy degradation in water vapor. *Radiat Res* **54**, 343-363 (1973).
6. Dingfelder, M., Inokuti, M. & Paretzke, H.G. Inelastic-collision cross sections of liquid water for interactions of energetic protons. *Radiat Phys Chem* **59**, 255-275 (2000).
7. ICRU. Stopping powers and ranges for protons and alpha particles. ICRU Report 49. 286 p. (International Commission on Radiation Units and Measurements, Bethesda, MD, 1993).
8. Friedland, W., Dingfelder, M., Jacob, P. & Paretzke, H.G. Calculated DNA double-strand break and fragmentation yields after irradiation with He ions. *Radiat Phys Chem* **72**, 279-286 (2005).
9. Tai, H. *et al.* Comparison of stopping power and range databases for radiation transport study. *NASA Technical Paper 3644*, 68 p. (Hampton, Virginia, 1997).
10. Curtis, S.B. Introduction to track structure and z^{*2}/β^2. https://three.jsc.nasa.gov/articles/Track-Structure-SCurtis.pdf (2016).
11. Stewart, R.D. *et al.* Rapid MCNP simulation of DNA double strand break (DSB) relative biological effectiveness (RBE) for photons, neutrons, and light ions. *Phys Med Biol* **60**, 8249-8274 (2015).
12. Höglund, E. DNA Fragmentation in Cultured Cells Exposed to High Linear Energy Transfer Radiation. Acta Universitatis Upsaliensis, Comprehensive Summaries of Uppsala Dissertations from the Faculty of Medicine 960, Uppsala (2000).
13. Höglund, E., Blomquist, E., Carlsson, J. & Stenerlow, B. DNA damage induced by radiation of different linear energy transfer: initial fragmentation. *Int J Radiat Biol* **76**, 539-547 (2000).
